# An exploration of the support received by mothers for kangaroo mother care practice along the health facility-community continuum in a sub-district of Northern Karnataka, India

**DOI:** 10.1371/journal.pone.0308738

**Published:** 2025-03-06

**Authors:** Maryann Washington, Leah Macaden, Prem K. Mony, Sumithra Selvam, Annetta Smith

**Affiliations:** 1 Division of Epidemiology & Population Health, St John’s Research Institute, Bangalore, India,; 2 Nursing Studies, School of Health in Social Science, The University of Edinburgh, Edinburgh, Scotland, United Kingdom,; 3 Division of Biostatistics, St John’s Research Institute, Bangalore, India,; 4 University of the Highlands and Islands, Scotland, United Kingdom; Makerere University School of Public Health, UGANDA

## Abstract

**Introduction:**

Early initiation with optimal duration of Kangaroo Mother Care (KMC), for all stable small babies (<2000grams at birth), is essential for accelerated reduction of neonatal mortality. The purpose of this paper is to explore the support received by mothers along the health facility-community continuum and its association with KMC practice.

**Methods:**

All live small babies aged >  4 weeks of life, who were residing in the Gangawati sub-district, were recruited on a rolling basis (Dec 2017-Sept 2018) to obtain the estimated sample size of 210. Mother-baby dyads were visited in their homes to collect information [knowledge, attitude, and support received] for KMC initiation and maintenance till required. Secondary data on KMC duration was obtained from the district-wide project database.

**Results:**

A total of 209 mothers with 227 small babies were interviewed (18 had twins). The mothers had a mean age of 23 (±4) years; and 7(±5) years of education, with 5 (±2) family members >  18 years in their households. More than half (51%) of the babies were female with a mean age of 35.6 (±7.5)days/ 4-6weeks and mean birth weight of 1693.6 (±221.4)grams irrespective of gestational age; 21.6% of whom were ≤ 1500g at birth. Most of the babies 205 (90.3%) were initiated on KMC at the health facility. The score obtained for KMC initiation [45%} and KMC maintenance support at the health facility [51.3%] was minimal. Multiple regression linear analysis showed that overall KMC support at the health facility was significantly higher for first-time mothers [β coefficient -1.54 (95% CI -2.87, -0.22)] and better knowledge scores on KMC [β coefficient 0.21 (05% CI 0.01, 0.42)]. KMC maintenance support was significantly higher for first-time mothers [β coeff -3.62 (95% CI -6.29, -0.96)] and for mothers whose babies had lower birth weights [β coeff -4.27 (95% CI -7.50, -1.05)].

**Conclusion:**

Mothers require support to initiate and continue KMC along the health facility-community continuum (S1 Table). The role of support at home would require further exploration to determine its association with KMC practice.

## Introduction

In India, Low Birth Weight (LBW) rate accounted for 16.4% of live births between 2015-16 [1]. This is of major concern since India ranked first for its contribution to LBW globally [2–3] with 25.5% of under-five mortality attributed to complications of prematurity and LBW [4]. Kangaroo mother care (KMC), an evidence-based practice is known to reduce mortality rates by 40% in small babies (*<2000grams at birth born either preterm or small for gestational age*) [5–6] and by 25% for sick babies between 1000-1799 grams compared to those who were stabilised and initiated on KMC later [7]. KMC thus offers a strategic solution to address this concern, and must be ensured as part of essential neonatal care (ENC) soon after birth for all stable small babies or soon after stabilisation of sick babies [6–7]; continued safely with optimal duration, under close supervision at the health facility and subsequently maintained for as long as possible at home. For this to be possible, it is assumed that mothers would require the support of health care workers (HCWs), community health workers (CHWs) and family members along the facility-community continuum [8–11] where care transitions take place for the newborn.

KMC, although operationally appearing to be simple to implement for stable small babies with benefits for the survival of small babies and their overall health, [6,7,12–14] has been limited in its implementation in low and middle-income countries including India [15], essentially forcing one to question the simplicity of its operationalization. Previous research reports lack of awareness, confidence, and support for mothers by HCWs and CHWs for low KMC coverage, [8–11]. Yet these studies did not describe what support mothers need to practice KMC.

Essentially KMC implementation includes {i} KMC initiation soon after birth for stable small babies or as early as possible after stabilization for a baby who is sick at birth [7,12,16]; {ii} Optimal KMC provision (*Skin-to-Skin contact [SSC] of ≥  8 hours daily with an ideal duration up to 24hours*) until the baby reaches 40 weeks of postmenstrual age or weight of 2500 grams (*presumably at 4-8weeks of unadjusted age*) [12,16]. Therefore, considerations of the plurality of birthplace and early voluntary discharge from the health facility within 24-48 hours by mothers after childbirth [17–19] even of small stable babies and the complex interplay between health systems requirements, organizational culture, and human behaviour that involves supportive and sensitive engagement between implementers (HCWs and CHWs) with mothers and family members [20–28] must be considered whilst planning supportive strategies for KMC uptake.

Behaviour is known to be influenced by social factors, affective factors, and rational deliberations[24–27]. Therefore, for “KMC practice along the health-facility community continuum” (outcome behaviour) to become the standard norm within this social context, an understanding of how mothers with their families could be supported is vital. A previous paper on determinants of KMC practice along the health facility community continuum did not show any effect of health facility preparedness on the duration of KMC but showed that KMC initiation support increased early initiation of KMC by 3% and KMC maintenance support at the health facility increased duration of KMC before discharge by 3% [29] This paper, thus reports details of the support provided to mothers at the health facility and at home and discusses how support to mothers needs to be operationalised along this continuum of care.

## Materials & methods

### Aim

To explore the KMC support received by mothers along the health facility-community continuum.

### Objectives

{i} Illustrate the KMC support as perceived by mothers for KMC practice along the health facility-community continuum.
{ii} Establish the association between KMC support with maternal characteristics and the characteristics of the babies.

### Methods

#### Design.

Operational research was chosen since it focused on developing solutions to problems identified towards implementation of a specific health program or service delivery component in this case KMC uptake with the healthcare system

#### Setting.

The study was conducted in the Gangawati sub-district comprising 145 villages. It is one of the four sub-districts of Koppal district, located in Northern Karnataka state. Gangawati has 15 public health facilities - three community health centers (CHCs), 11 primary health centers (PHCs), one sub-district hospital; 12 childbirth facilities, and six Level I or II neonatal care units that are privately managed.

#### Participants.

Mothers with small babies, and foster KMC (fKMC) providers

#### Sampling technique.

Consecutive non-probability sampling technique was used for the selection of small babies by rolling enrolment. Small babies were eligible for recruitment if they were born or transferred to a health facility in the sub-district and had survived 4-weeks of life (unadjusted age). Mothers and fKMC providers of the eligible small babies were automatically recruited if they resided in the sub-district with their small babies (aged between 4-6 weeks of life) and were available when the investigator and research assistant made home visits.

#### Sample size.

A sample of 175 small babies was calculated with a relative precision of 15% and 95% confidence interval (CI) based on the {i}coverage of stable small babies with KMC increasing from < 2% in Koppal sub-district, to 5% within the three months from the start of the district-wide project (August – October 2016); {ii} findings and evidence from a previous study [30] and {iii} predictions that KMC coverage would improve to 40% by the end of a year. After accounting for 20% attrition, the revised sample size was estimated at 210 small babies and their mothers. One fKMC provider was selected from each family with a preference for a male over a female provider if more than one fKMC was available, to obtain a balanced representation of knowledge, attitude, and support received for the provision of KMC from both genders.

### Key variables

- *Knowledge* of mothers and fKMC providers on KMC referred to the score obtained based on their responses to items on general aspects of KMC, its benefits, and monitoring of a baby when being provided KMC.
- *Attitude* of mothers and fKMC providers referred to the score obtained based on the responses to related items on the questionnaire.

Knowledge and attitude were presumed to be reflective of support received for KMC practice.

- *Support* included {i} KMC initiation support at the health facility referred to the score obtained based on the mothers’ responses to items related to support received such as information/ counselling on KMC and assistance provided to the mother for positioning the baby with SSC; {ii} KMC maintenance support at the health facility referred to the score obtained based on mothers’ responses to items related to direct help the mother received for providing KMC from the HCWs, fKMC provider, or peer mothers (*those who were confident and had experience with providing KMC at the health facility*), access to a KMC kit (which consisted of *a KMC bag to secure the baby, cap, socks and diaper for the baby and a binder to safely position the baby, including a palada – a beaked metal feeding cup*) at the health facility. KMC initiation support and maintenance support at the health facility were combined to provide the score for KMC support at the health facility. {iii} KMC maintenance support at home referred to the score obtained based on mothers’ responses to items on support they received at home from CHWs, from fKMC providers for the provision of SSC, and from other family members for domestic chores or childcare. The knowledge, attitude, and support score obtained by fKMC providers was added to KMC maintenance support at home, presuming that these would impact their provision of support to the mother at home.

#### Data collection tools.

An investigator-developed validated questionnaire was used to assess retrospectively the support received for KMC practice with open-ended items. The questionnaire contained 11 items to assess knowledge of KMC for both mothers and fKMC providers, with each item, scored against a predetermined scoring key based on the number of open appropriate responses provided. Thus, the maximum possible knowledge score was 30. There were four items used to assess the attitude of mothers and fKMC providers towards KMC, totaling a maximum possible score of 4.

While assessing KMC support for mothers, since there were no standardized tools available, key items were developed by the investigator and validated by eight external experts based on what was reported in the literature as support from the HCWs [31–33] and home through family members and spouse [10,12,19,21,26]. KMC support was categorized as KMC initiation support at the health facility, KMC maintenance support at the health facility, and KMC maintenance support at home. Twelve items in the questionnaire for mothers helped to arrive at scores of 14 for KMC initiation support at the health facility, 15 for KMC maintenance support at the health facility, and 38 for KMC maintenance support at home (Table 1). Scoring for the support received was developed by the investigator [MW] based firstly on their response to the closed-ended item of whether they received any support. E.g. they were asked “Who helped you to start KMC in the hospital?” the responses included “No one, it was not started in the hospital, nurse, nurse mentor, counsellor, doctor, other mothers, any other”. If they answered “No one” or not started in the hospital”, a score of 0 was given. While if they answered either nurse, counsellor or other mothers, then score was allotted accordingly (See Table 1). Thus for example when the highest number of open responses obtained from mothers was six, then a maximum score of seven [1 + 6 = 7] was assigned to the given item (See Table 1). Alternatively, scoring was based on the weightage the item had, for example, “if a mother mentioned that she had an fKMC provider, the weightage was given as 5” as this was considered “essential criteria” for support. This scoring system was validated by the three supervisors (LM, AS, PM) and the statistician (SS) iteratively. The scores of fKMC providers’ knowledge, attitude, and support received were categorized based on the percentage obtained, and these categories were allotted a score and placed under “KMC maintenance support at home” as given in Table 1.

**Table 1:** Scoring system - support for KMC practice at the health facility or at home.

Item in tool	Possible responses	Components of support for KMC	Scoring system	Maximum possible score allotted
***KMC initiation support at health facility***				***14***
Mother: Q13 Who gave you information about KMC in the hospital	No one □ Yes Nurse □ Nurse mentor □ Counsellor □ Doctor □ Other mothers □ Any other □	Counselled/Informed about KMC By whom?	0 = None, not started; 1 = Yes Plus 1 = for each open-response provided	7
Mother: Q12 Who helped you start KMC in the hospital?	No one □ It was not started in the hospital □ Yes Nurse □ Nurse mentor □ Counsellor □ Doctor □ Other mothers □ **Any other**	Assistance for KMC initiation By whom?	0 = None/ not started; 1 = Yes, Plus 1 = for each open-response provided	7
***KMC maintenance support - health facility***				***15***
Mother: Q14. Who helped you most in the hospital to give KMC?	No one □ Yes Nurse □ Nurse mentor □ Counsellor □ Doctor □ Other mothers □ Any other □	Person providing the most help Who?	0 = None; 1 = Yes, Plus 2 = 1-2 persons 3 = 3-4 persons 4=>4 persons	5
Mother: Q15. Did you receive a KMC Kit from the hospital?	Yes □ No □	Provision of KMC Kit	0 = No 5 = Yes	5
fKMC: Q18a did you provide KMC in the hospital	Yes □ No □	Availability of fKMC provider	0 = No 5 = Yes	5
***Support for KMC maintenance at home***				***38***
Mother: Q18. Who helped you most at home to continue KMC	Mother □ Father □ Sister □ Spouse □ CHW □ Any other □	Person providing the most help Who?	0 = No support 3=≥1 persons	3
Mother: Q18. Number of people available to help mother at home	Based on the above responses	Number of persons available to support mother at home	0 = No support 1 = 1 person 2 = 2 persons 3 = ≥ 3 persons	3
Mother: Q17 How did the CHW help you?	Did not help at all □**/** Did not visit at all □ Gave information on KMC □ Checked weight of baby □ Helped find ways to increase KMC hrs □ How to monitor a baby on KMC □ Any other (about feeding, etc.)	Support from the CHW as reported What support was provided	0 = No support 1 = Yes Plus 1 point for each response	6
fKMC: Q18	fKMC provider available	fKMC provider available *(yes or no)*	0 = No fKMC provider 5 = fKMC available	5
fKMC: Q5	Daily duration of SSC	Daily duration of SSC provided by fKMC provider:	0 = No fKMC provider 2 = Yes -fKMC provider, but not available 3 = Yes, 2hours/day 4 = Yes, 3-4hours/day 5 = Yes, 5-6hours 6 = Yes, > 7hours	6
fKMC: Q6-Q11, Q13-16	meaning, who can provide, requirements, duration, benefits monitoring	fKMC knowledge score (maximum score 30 points)	0 = No fKMC provider 1 = Yes fKMC provider, but not available 2 = ≤ 25% of score 3 = 26-50% of score 4 = 51-75% of score 5 = > 76% of score	5
fKMC: Q12, Q19, Q21, Q22		• fKMC Attitude score (*maximum score 4 points*)	0 = No fKMC provider 1 = Yes, fKMC provider but not available 2 = ≤ 25% of score 3 = 26-50% of score 4 = 51-75% of score 5 = > 76% of score	5
fKMC: Q17 Who gave you information on KMC in the hospital	No one □ Nurse □ Nurse mentor □ Counsellor □ Doctor □ Other mothers □ Any other □	fKMC KMC support score *Maximum score = 18* *(Q17 = 7 score* * Q18 = 7 score;* *Q20 = 4 score [one for* *each point*)	0 = No fKMC provider 1 = Yes, fKMC provider but not available 2 = ≤ 25% of score 3 = 26-50% of score 4 = 51-75% of score 5 = > 76% of score	5
fKMC: Q18 Who helped you most to provide KMC in the hospital	No one □ Nurse □ Nurse mentor □ Counsellor □ Doctor □ Other mothers □			
fKMC:Q20 Help provided by CHW	Did not help at all □ Gave information on KMC □ Checked weight of baby □ Helped find ways to increase KMC hrs □ How to monitor during KMC □			

Mother: Q: Refers to the Question Number in the questionnaire for the Mother

fKMC: Q: Refers to Question Number in the questionnaire for the fKMC provider

Knowledge, attitude and support score of fKMC provider added under “KMC maintenance support at home”

### Data collection

Mothers were identified from the list of all small babies available in the district-wide project database, starting with babies born in December 2017 till the sample size was reached in September 2018. From this list, 160 of 408 (39.2%) small babies were not eligible for recruitment either because: {i} they did not survive [47/408 -11.5%]; were referred out of the district for clinical management [33/408- 8.1%; of whom three did not survive 4-weeks of life] or were discharged against medical advice within three days of life [7/408-1.7%]; {ii} they were residing out of the sub-district [73/408 – 17.9%]. From the remaining 248 of 408 (60.8%) who were eligible for recruitment, [One baby did not survive 4 weeks of life], it was possible to recruit 227 small babies for this study, as 21 were not available despite two successive visits to their homes within one week [31]. The investigator along with a local field investigator visited the 209 mothers with their baby/babies in their homes (18 mothers had twins).

Only 21% (44/209) and 47.3% (99/209) of mothers, had the support of fKMC provider/s at the health facility and home, respectively. Of all available fKMC providers, 84% (83/99) had completed the questionnaire. Attrition of eligible fKMC providers resulted due to {i} return to employment - 11% (11/99); {ii} caring for hospitalised family members -2% (2/99); {iii} return to their usual place of residence - 2% (2/99); {iv} hearing impairment - 1% (1/99).

#### Data Analysis.

Descriptive statistics were reported as mean (±standard deviation) or median with interquartile ranges (IQR) for continuous variables when data was not normal, number, and frequencies for the categorical variables. Univariate and multiple linear regression analysis was performed to determine the association of maternal and baby characteristics with KMC support at the hospital and KMC maintenance support at home. Variables that were significant and those with p-values up to 0.20 in the univariate analysis were considered for multivariable analysis. All the analyses were performed using SPSS version 25 and STATA 15.0.

### Ethics approval

Ethics approval was obtained from the St Johns Medical College and Hospital, Institutional Ethics Committee (Ref No 64/2017) in April 2017. Approval was also obtained from the NHS Invasive or Clinical Research (NICR 16/17-Paper 48) committee in May 2017 for the study period. Written consent was obtained from the mother and fKMC provider. Data was anonymised for analysis and was overseen only by the investigator and district-wide project staff. Since this study was nested within the district-wide study which was registered with the Clinical Trials Registry of India (CTRI/2017.07.008988), a separate registration was not considered essential.

## Results

The mean age of small babies was 35.6 (±7.5) days/ 4-6 weeks with a birth weight of 1693.6 (±221.4) grams (Table 2). The median (IQR) duration of hospitalization for the babies was 4.57 (5) days, with a range of 1-30 days. More than a quarter of babies were initiated on KMC on day-1 of life [28.7% (64/223)] and nearly a third of them were started on day 2-3 of life [30.9% (69/223)] respectively, with the median day of KMC initiation being 3 (5) days [31]. The median duration of KMC increased from [6.0 (7.0)] hours on the day of initiation to [8.0 (7.2)] hours on the day before discharge and then reduced to [6.0 (7.3)] hours on the 7^th^ day after discharge [31]. KMC was provided for 30.2 ± 8.5 days with a range of 2 to 45 days [31].

**Table 2:** Characteristics of small babies.

Characteristics of small babies	
**Birth weight (grams) -** Mean (±SD)	1693.6 (±221.4)
**Sex [No (%)]**	**(n = 227)**
- Male	94 (41.4%)
**-** Female	133 (58.6%)
**Status at birth [No (%)]**	**(n = 227)**
- Well	37 (16.3%)
** -** Sick	190 (83.7%)
**Place of birth [No (%)}**	**(n = 227)**
- Sub-district hospital	79 (34.8%)
- CHCs/PHCs	50 (22.0%)
- Private	78 (34.4%)
- Home	20 (8.8%)
**Place of hospitalisation [No. (%)]**	**(n = 227)**
- Sub-district hospital	69 (30.4%)
- CHC/PHCs	36 (15.9%)
- Private	122 (53.7%)
**Duration of hospitalisation [No. (%)]**	(n = 226)^©^
- ≤ 3 days	102 (45.1%)
- 4-7 days	53 (23.5%)
- 8-14 days	55 (24.3%)
- > 14 days	16 (7.1%)

*©: One mother could not recollect the duration of hospitalisation*

### Characteristics of mothers and fKMC providers

The mean age of mothers and fKMC providers was 23.5 (±4) years and 36.9 (±13.9) years, respectively. Nearly a third of the mothers [64% (134/209)] and most of fKMC providers [78% (65/83)] had ≤ 8^th^ grade education (Table 3). Fifty-five percent (114/209) of the mothers were first-time mothers.

**Table 3:** Characteristics of mothers with small babies, fKMC providers.

Characteristics of mothers and fKMC providers	Mothers (n = 209)	fKMC providers (n = 83)
**Age (years)**		
- Mean (±SD)	23.5 (±4) years	36.9 (±13.9) years
**-** Range	17-35 years	16-70 years
**Education [No. (%)]**		
- ≤ 8^th^ grade	134 (64.0%)	65 (78.3%)
- > 8^th^ grade	75 (36.0%)	18 (21.7%)
**Occupation [No. (%)]**		
- Skilled workers	18 (8.6%)	6 (7.2%)
- Unskilled workers	109 (52.2%)	51 (61.5%)
- Homemakers	82 (39.2%)	26 (31.3%)
**Number of children** [No. (%)]		Question not included for fKMC provider
- 1	114 (55%)	
- 2	61 (29%)	
- ≥ 3	34 (16%)	

### Knowledge, attitude of mothers and fKMC providers on KMC

The knowledge score of mothers and fKMC providers (Table 4) on general aspects of KMC was good [12.4/16 and 12.3/16 respectively (>75%)], but below average [3.2/9 and 2.9/9 respectively (<35%)] on benefits of KMC and monitoring a baby receiving KMC [1.7/5 and 1.6/5 respectively (<35%)]. Both had extremely favourable attitudes towards KMC (Table 4).

**Table 4:** Knowledge of mothers and fKMC providers 4-6 weeks after the birth of the small baby.

Knowledge domains	Knowledge score		
	Maximum score	Mothers (n = 209)	fKMC providers (n = 83)
		Mean (±SD)	Mean ( ± SD)
- General knowledge of KMC	16	12.4 (±1.6)	12.3 (±1.6)
- Benefits of KMC	9	3.2 (±1.4)	2.9 (±1.3)
- Monitoring a baby while providing KMC	5	1.7 (±1.1)	1.6 (±1.1)
*Overall knowledge of KMC*	30	17.3 (±3.2)	16.8 (±3.1)

Mothers with > 8^th^ grade had higher knowledge scores compared to those with ≤ 8^th^ grade education (18.2 ± 3.2 vs 16.9 ± 3.1; p < 0.05) and those with unskilled work (16.7 ± 3.1), had significantly lower knowledge scores at 0.05 level than those who were homemakers (17.8 ± 2.9) or engaged with skilled work (19.3 ± 3.5). The median attitude score of both mothers and fKMC provider was 4.0.

Most of the fKMC providers [77% (76/99)] were women (maternal mother, mother-in-law, sister-in-law or sibling). The remaining fKMC providers were men who included 15% spouses and 8% other male relatives.

Nurses/health assistants were the primary HCWs who counselled/informed 81% (159/196) of mothers and 78% (45/58) of fKMC providers on KMC initiation (Table 5). Nurses/health assistants primarily assisted most mothers, [84% (157/209)] to position the baby KMC initiation; and supported mothers to maintain KMC at the health facility [67% (125/187)].

**Table 5:** Details of KMC support received by mothers and fKMC providers along the health facility-community continuum.

KMC initiation support at the health facility	Mothers	fKMC providers
***Counselled/ informed about KMC by*** [No. (%)^**^]	n^a^ = 196^a^	n^b^ = 58
- Nurse/ health assistant	159 (81%)	45 (8%)
- Nurse mentor	65 (33%)	16 (28%)
- Counsellor	12 (6%)	2 (3%)
- Doctor	66 (34%)	11 (19%)
- Peer mother	54 (28%)	16 (28%)
- Audio - visual aids	10 (5%)	4 (2%)
***Assistance for KMC initiation provided by*** [No. (%)^**^]	n = 209	Question not included for fKMC providers
- Nurse/health assistant	157 (84%)	
- Nurse mentor	69 (44%)	
- Counsellor	4 (3%)	
- Doctor	60 (38%)	
- Peer mother	40 (26%)	
**KMC maintenance support at health facility**	Mothers	fKMC providers
***Persons providing most support*** [No. (%)^**^]	n^c^ = 187	n^d^ = 48
- Nurse/ Health Assistant	125 (67%)	35 (73%)
- Nurse mentor	55 (29%)	12 (25%)
- Counsellor	3 (2%)	2 (4%)
- Doctor	15 (8%)	2 (4%)
- Peer mother	28 (15%)	10 (21%)
***Provision of KMC kit*** [No. (%)]	n = 209	Question not included for fKMC providers
* -* Yes	157 (75%)	
- No	52 (25%)	
*Availability of f****KMC*** *provider at facility*	N = 209	
- Yes * -* No	44 (21%) 165 (79%)	
**Support for KMC maintenance at home**		
***Provided by CHWs* [No (%)** ^**^]	n^f^ = 208	n^g^ = 79
Information on KMC	201 (97%)	79 (100%)
Checked weight	185 (89%)	67 (85%)
Helped find ways to increase KMC	129 (62%)	53 (67%)
Gave information on how to watch the baby while on KMC	19 (9%)	5 (6%)
Referred baby to facility for a health issue	25 (12%)	3 (4%)
***Person who supported mother at home* [No (%)** ^**^]	n^h^ = 191	Question not included for fKMC providers
Maternal mother/ Mother- in- law	163 (85%)	
Maternal father	8 (4%)	
Sister/ Sister- in- law	59 (31%)	
Spouse	24 (13%)	
CHW	25 (13%)	

** *multiple responses from mother or fKMC provider and hence no (%) more than total number (100%).*

*a-h: subset of mothers or fKMC providers who responded affirmatively to the question.*

Except for one, all other mothers and all fKMC providers received support from CHWs at home (Table 5) through either provision of information on KMC [97% (201/208) and 100% (79/79) respectively] or through finding ways to increase the daily duration of KMC [62% (129/208) and 67% (53/79) respectively].

### KMC support as perceived by mothers

The KMC initiation support [45% (6.3/14)] and KMC maintenance support at the health facility [51.3% (7.7/15)] of mothers was minimal (Table 6). Of all the domains of KMC maintenance support at home, the domain “*Support with domestic chores*” was the highest, [96.7%, 2.9(±0.36)].

**Table 6:** KMC support score of mothers along the health facility community continuum.

KMC support at the health facility	Support score of mothers (n = 209)		
*KMC initiation support*	Maximum score	Mean (±SD)	Median (IQR)
- Counselled/informed about KMC	7	3.3 (±1.2)	3 (3)
- Assistance to initiate KMC	7	3.0 (±1.3)	3 (3)
*Subtotal KMC initiation support*	14	6.3 (±2.3)	6 (2)
***KMC maintenance support***			
- Person providing the most support	5	2.8 (±1.1)	3 (3)
- Provision of KMC kit	5	3.8 (±2.2)	5 (5)
- Availability of fKMC provider	5	1.1 (±2.0)	0 (0)
*Subtotal KMC maintenance support*	15	7.7 (±3.7)	8 (1)
***Total KMC support at the health facility***	29	13.9 (±5.2)	14 (4)
**KMC maintenance support at home**	**Score of mothers (n = 209)**		
	**Maximum Score**	**Mean (±SD)**	**Median (IQR)**
* -* Support with household chores	3	2.9 (±0.36)	3 (0)
* -* Number of persons to support the mother	3	1.4 (±0.72)	1 (1)
* -* fKMC provider available at home	5	2.4 (±2.50)	5 (5)
* -* Daily KMC duration by fKMC provider	6	1.7 (±2.01)	0 (4)
* -* Knowledge of fKMC provider	5	1.6 (±1.84)	1 (4)
* -* Attitude of fKMC provider	5	2.0 (±2.34)	1 (4)
* -* Support for fKMC provider	5	1.3 (±1.52)	1 (3)
* -* Support from CHW	6	3.7 (±0.88)	4 (1)
***Total KMC maintenance support at home***	38	16.9(±10.25)	13 (20)

The number of children and knowledge of KMC of the mothers was significantly related to the KMC support received at the health facility (Table 7), while the number of children and birth weight of the baby was significantly related to KMC maintenance support at home (Table 8).

**Table 7:** Univariate and multivariable analysis on KMC support at health facility with maternal and baby characteristics.

Characteristics	Univariate Analysis		Multivariable Analysis	
*Maternal*	Beta coefficient	95% Confidence interval	Beta coefficient	95% Confidence interval
Age (years)	-0.11	-0.28, 0.06		
Education	0.34	-1.01, 1.68		
Occupation	0.46	-0.16, 1.08		
Number of children	-1.56	-2.89, -0.23	-1.54	-2.87, -0.22
Knowledge on KMC	0.22	0.01, 0.42	0.21	0.01, 0.42
*Babies*				
Birth weight of baby	-1.06	-2.68, 0.56		
Sex of baby	-0.44	-1.8, -0.91		

*KMC initiation support at the facility and KMC maintenance support at the facility combined.

**Table 8:** Univariate and multivariable analysis on KMC maintenance support at home with maternal and baby characteristics.

Characteristics	Univariate Analysis		Multivariable Analysis	
	Beta Coefficient	95% Confidence Interval	Beta Coefficient	95% Confidence Interval
*Maternal* Age (years)	-0.06	-0.40, 0.29		
Education	0.67	-2.05,3.40		
Occupation	0.14	-1.12, 1.39		
Number of children	-3.34	-6.04, -0.64	-3.62	-6.29, -0.96
Knowledge on KMC	0.09	-0.32, 0.51		
*Babies*				
Birth weight of baby	-3.92	-7.18, 0.66	-4.27	-7.50, -1.05
Sex of baby	0.80	-1.95, 3.56		

## Discussion

KMC support as perceived by the mother along the health facility-community continuum is discussed under two headings, KMC support at the health facility and KMC support at home.

### KMC support at the health facility

KMC is a behaviour that needs to be understood, learned, accepted, and practiced as part of the ENC of small babies. Early initiation of KMC of stable or sick small babies soon after birth is known to improve their survival and reduce morbidity [6–7]. The KMC initiation support although minimal (45% of the total score), was known to impact the early initiation of KMC[31]. Nurse mentors, who were project staff were mentioned by more than a third of the mothers as professionals who counselled and assisted in initiating KMC. Thus for the sustainability of this practice, it is important to consider the following whilst planning KMC implementation at the health facility [7,10,34]: {i} HCWs need to integrate and provide information on KMC to mothers/family members as part of their routine service. {ii} HCWs need to help mothers position their small babies safely. Given the physiological and psychological benefits of KMC [14,28], and the possibility of experiencing a restorative process by just having their small baby close to them while practicing KMC [9] it would be pragmatic that HCWs, specifically nurses, health assistants, and doctors to provide information and help mothers to initiate KMC as early as possible, for stable small babies. Peer mothers were also identified as key resources by more than 25% of mothers for informing them about KMC or assisting them in initiating KMC. HCWs are well placed to identify peer mothers who are confident in providing KMC to assist new mothers in the health facility especially when they are challenged by workforce shortage and heavy workload. We did not explore whether the practice of SSC was initiated at the birth of the small baby in this study. Yet it is a strategy that could be recommended to ensure early initiation of KMC. Exploration of how SSC at birth could transition to maintenance of KMC was not fully explored since this wasn’t within the scope of this study.

Despite the shortened hospitalization after childbirth and minimal KMC maintenance support at the facility (53% of total score), KMC duration had increased before discharge indicating that mothers were either motivated to practice KMC for optimal duration or did so to go home early. Yet, one must be mindful that mothers especially those with twins or even first-time mothers would need time, assistance, support, and confidence to position their small babies (1013-1990grams) safely and comfortably, monitor them while on KMC and sustain the provision of ≥ 8 hours daily KMC before discharge from the health facility. First-time mothers were provided significantly more support at the health facility than mothers with more than one child. HCWs must be cautioned to support all mothers, especially in the first few days, so that the small babies are safely positioned. This study showed that mothers’ knowledge was significantly related to KMC support at the health facility, possibly due to the number of HCWs who were in contact with the mother-baby dyad at the health facility. Health managers need to take a cue from this study to reinforce with HCWs that each contact with mothers and family members could be a prime opportunity to provide information on the benefits of KMC and how to monitor a baby while on KMC; however, this needs to be offered in simple and jargon-free language. Some mothers reported that they received information on KMC via audio-visual aids such as posters, brochures, videos, etc., available in the health facility. This finding reiterates the importance of using multiple resources such as HCWs, peer mothers, and audio-visual aids to reinforce information on KMC to enhance uptake, especially in the context of low educational status of mothers. Although knowledge of mothers was not identified as a determinant of KMC practice [29], the awareness of its benefits could facilitate more confidence in first-time mothers and those from a lower education level.

KMC maintenance support at the health facility was measured by the number of people who helped the mother, the presence of a fKMC provider, and the provision of a KMC kit that would assist the mother in positioning the baby safely for KMC. There were at least 2-5 people [mostly HCWs] who supported the mother in the health facility. Further, the availability of fKMC providers at the health facility was minimal. Typically within this south Indian setting, mothers return to their parents’ home sometime before and following childbirth and tend to remain there for 3-5 months depending on whether they had a normal or cesarean delivery. Family members are important assets in such a cultural context who can adopt the role of fKMC providers. Orientation of prospective mothers and their families as part of antenatal care seems to be an untapped potential (although not explored in this study) to increase participation and confidence among family members to become fKMC providers. It would also require health facilities to have an open visitation policy to enable key family members to support the mother. However, if this opportunity was missed, then HCWs should identify and counsel one or two family members on KMC at the birth of a small baby, with targeted attention of those mothers with twins, to facilitate further increase of KMC maintenance support [9–11]. A previous study showed that even one lesson on KMC was sufficient for mothers to want to adopt KMC [34] while another study demonstrated that KMC initiation and KMC maintenance support at the health facility significantly impacted early initiation of KMC and duration of KMC before discharge [29]. One might argue that HCWs are challenged with workload due to workforce shortage, yet supportive strategies that improve their capacity [11,20,28,30,35] to provide key messages on KMC to mothers and families effectively could be vital in further enabling them, to ensure mothers are informed. Advocating for KMC could be to their advantage [in terms of staff time given the workforce shortage], as mothers and families begin to care for the baby in the health facility [36]

The support HCWs provided at the health facility could have possibly influenced the awareness and attitude of mothers and fKMC on KMC. Credibly their knowledge was average (just above 50%), with extremely favourable attitudes towards KMC, even a month after childbirth. Yet, attention must be paid to providing information to them on the benefits of and how to monitor a baby while on KMC. This information could be reinforced through multiple information resources such as the provision of a pamphlet or brochure, use of mHealth, and even by CHWs after discharge. Unequivocal coordinated guidance from all the HCWs and peer mothers at the health facility on KMC maintenance is essential for its safe provision.

### KMC maintenance support at home

KMC maintenance support at home post-discharge from the health facility comprised of a package of support by family members for domestic chores, support from fKMC providers to increase SSC, and finally support from CHWs. The CHWs had a significant role in the provision of support and were acknowledged as vital for sustaining KMC practice at home by most mothers and fKMC providers. The fact that the percentage of fKMC providers had increased from 21% at the health facility to 47.3% at home, could be probably attributed to CHWs. This assumption is made since more than 97% of mothers and fKMC providers mentioned that CHWs had informed them about KMC and more than two-thirds of them mentioned that the CHWs had helped them to find ways to increase KMC duration. Although KMC maintenance support at home was reported as insignificant in facilitating the increased duration of KMC [29], it must be considered crucial for the mother to continue KMC for the recommended duration. Mothers and family members thus, {i} might need to be reminded of the benefit of ≥ 8 hours KMC duration which could be achieved if they all shared and coordinated the responsibility {ii} require time to re-adjust to the environment of home with a small baby; {iii} would need support to re-acclimatise to their routine with domestic chores, childcare, and attending to visitors in the first few days post-discharge, in addition to providing KMC {iv}would probably need focused breastfeeding counselling so that they continue to exclusively breastfeed their babies[37]. CHWs role in facilitating family members to be fKMC providers to support the mother, [17,38] could be reinforced through micro-planning tools that help them address possible barriers to KMC practice with the family, or help them underline the significant benefits of prolonged KMC duration on the neurodevelopment of the small baby, especially if they had not started this role at the health facility. CHWs would also need to reinforce information on the importance of breastfeeding or breastmilk feeds for these babies. Increasing awareness of family members and the community on the need to continue KMC and how they could be of assistance to ensure the baby gets more than 8 hours of KMC daily is essential. This support is ever more pressing, with the view that mothers are young, mostly first-time mothers, with lower education levels and thus might be hesitant to request for support and assistance from family members for KMC provision. Credibly, KMC maintenance support at home in this study was significantly better when the birth weight of the baby was lower and for first-time mothers.

The role of CHWs in supporting KMC practice in this study included visiting mothers at home, reinforcing information on KMC and how to monitor the baby while providing KMC, finding ways collaboratively with the mother to increase SSC duration, checking the weight of the baby or referring the baby to a health facility. Given the short duration of hospitalisation, the critical role played by CHWs in KMC practice has been highlighted in this study. Most of the KMC was provided at home, and this could have been primarily due to the support mothers had from family members and CHWs. Overall, these findings typically indicate that mothers require additional support at home, especially in the first week post-discharge to sustain the optimal duration of KMC, given that KMC duration had reduced in this cohort of babies from the day before discharge to the 7^th^ post-discharge day. Hence, in this cultural context where there were more than 5 adult family members at home, it could be worthwhile to explore ways by which they all could play a role in increasing the hours of SSC and ensuring the mother exclusively breastfeeds her baby.

## Limitations of the study

This study was limited to only babies who were available and had survived 4-weeks of life in the sub-district. The babies excluded from the study could have varied by characteristics, for example – health status at birth or birth weight are known confounders for KMC practice and would thus require further exploration. The validated investigator-developed scoring system to quantify KMC support at the health facility or home was not tested rigorously, as this was beyond the scope of the study. Yet, the results seem logically plausible given that first-time mothers received more support both at the health facility and at home. Moreover, support was measured 4-6 weeks after initiating KMC, and hence the chance of recall bias is a possibility.

## Implications of the findings

The findings of this study on extrapolation, point to the need for support to enable optimal KMC practice for a small baby at three crucial points – before childbirth, at childbirth and after discharge from the health facility (Table 8). The information must be provided to all pregnant women along with significant family members about KMC and its benefits and this must be reinforced at the childbirth of a small baby. Building awareness of family members might help them decide how they could assist the mother in achieving the needed hours of SSC. Health facility managers would need to ensure an open visitation policy for family members. This would help HCWs to identify and equip family members as fKMC providers with knowledge, appropriate attitudes, and skills for KMC practice at the health facility. Multiple KMC information resources must be available in the health facility to improve awareness of mothers and family members on KMC who could thereby demand it. The KMC kit that contains materials to position the baby safely for KMC must be available, or mothers could be taught how to improvise and use materials at home – such as a shawl/ soft cotton saree to ensure the baby is safe and secure during KMC provision.

KMC includes both SSC and exclusive breastfeeding. Hence a focused breastfeeding programme directed towards increased SSC, frequent breastfeeding, good positioning, and enhanced involvement of the father known to improve short and long‐term (6 months) breastfeeding success is deemed critical in a scenario of shortened hospitalisation [37]. HCWs should reinforce to mothers that both components, SSC and exclusive breastfeeding are equally important for improved survival and well-being of the small baby [17]. Additional support for first-time mothers and those with twins is needed for them to learn the skill of expressing breastmilk and gain confidence in feeding the baby successfully before discharge from the health facility.

CHWs and family members would need to provide additional support once the mother-baby dyad is discharged from the health facility. The fact that mothers were discharged early from the health facility, points to the need, to improve the capacities of CHWs to support mothers with providing effective KMC for as long as necessary.

## Conclusion

This study clearly highlights the components of support that mothers could receive both for KMC initiation and maintenance at the health facility. Given that the duration of hospitalisation is limited for a considerable proportion of small babies, it is crucial that mothers are further supported at home through CHWs and family members. There is no doubt that mothers are motivated and inclined, able to learn how to provide KMC in a brief time, are willing and keen to practice KMC.

## Supporting information

S1 Table Standard Operating Procedure – Provision of support for KMC practice along the health facility – community continuum.
(DOCX)

S1 Data KMC support data. (PDF)

S2 Data KMC support data coding sheet. (PDF)

## Abbreviations

CHCs: Community Health Centres
CHWs: Community Health Workers
ENC: Essential Newborn Care
fKMC: Foster KMC
HCWs: Health Care Workers
KMC: Kangaroo Mother Care
LBW: Low Birth Weight
PHCs: Primary Health Centres
SDHs: Sub-District Hospitals
SNCUs: Special Newborn Care Units
SSC: Skin-to-Skin Contact.
