# Supplementary material for: An exploration of the support received by mothers for kangaroo mother care practice along the health facility-community continuum in a sub-district of Northern Karnataka, India
Source: PLoS One. 2025 Mar 6;20(3):e0308738. doi: 10.1371/journal.pone.0308738 (PMC11884690; doi:10.1371/journal.pone.0308738)
Supplement: S1 Table — (DOCX) [file pone.0308738.s001.docx]

|  | **S1: Standard Operating Procedure – Provision of support for KMC practice along the health facility – community continuum** | | |
| --- | --- | --- | --- |
|  | **Community** | **Health facility** | **Community** |
| **Context** | Antenatal Care | Pregnancy + Childbirth + Hospitalisation of the LBW baby | Continuation of KMC for 4-6 weeks at home |
|  | **CHW’s role** | **HCW’s role** | **CHW’s role** |
| Input | - Educate and counsel mothers and the community at large on KMC and exclusive breastfeeding, - Identify potential fKMC providers. | - Educate and counsel mothers at antenatal visits on effective KMC, - Assistance and supervision from all HCWs including peer mothers’ to safely position the baby for KMC in the initial days, - KMC kit for mothers to initiate and maintain effective KMC, - Recognise HCWs who promote effective KMC as champions, - Ensure fKMC providers are identified and begin supervised KMC provision at the health facility before discharge. | - Provide support through daily home visits after discharge for a week to   - Identify barriers and solutions collaboratively with the family to enhance the practice of effective KMC,   - Encourage fKMC providers to provide KMC or assist with domestic chores,   - Reinforce the need for exclusive breastfeeding,   - Remind mother to continue KMC while breastfeeding, |
| DHOs’ role | **Ensure community mobilisation for KMC through multiple methods – street plays, drama, local TV programs, posters & billboards**  **Ensure an open visitation policy to the SNCU or NBSU to encourage the practice of fKMC providers**  **Incentivise CHWs for daily home visits for seven days in the event of a small baby** | | |
| Resources & systems requisites |  | - KMC information materials: brochures/videos/posters - Ensure link cards are provided to mothers on discharge so that CHWs are informed of the need to visit small babies at home. | - Small baby helpline in the event of any danger signs - Referral of babies with any danger signs. |
