# Supplementary material for: An exploration of the support received by mothers for kangaroo mother care practice along the health facility-community continuum in a sub-district of Northern Karnataka, India
Source: PLoS One. 2025 Mar 6;20(3):e0308738. doi: 10.1371/journal.pone.0308738 (PMC11884690; doi:10.1371/journal.pone.0308738)
Supplement: S1 Data — (PDF) [file pone.0308738.s002.pdf]

An exploration of the support received by mothers for KMNC practice along the health facility continuum an operation research study in a sub-district of northern Karnataka India – Part2

This document provides the labelling for all the variables reported in the paper.

**Variable Name**	**Variable labelling**
PID	Participant identification Number
S. No	Serial Number
Q4.BirthWeightGrams	Birth Weight in Grams
BWcat	Birth weight category 1 <=1500, 2 =>1500
Q5.SexM1F2O0	Sex of the baby 1=Male , 2=Female
Q2.PlaceofBirthDHTH1CHC2PHC3Pvt4Home5OD5	Place of Birth 1= DH and TH, 2=CHC, 3=PHC 4=Pvt<br>5=Home 6= out of district
placebirth	Place of birth
Q8.AdmittedWhereTH1CHC2PHC3Pvt4Referredout5at home0	Admitted<br>1= DH and TH, 2=CHC, 3=PHC 4=Pvt<br>5= Referred out, 0=at Home
Admitted	Category for admission Public and private
babystatusKMC2Well1Sick2greenNullatBirth	Baby status 2= Well, 1= Sick
Q1.DOB	Date of birth
M_Q11.ii. DateKMCstartedMothers	Date of KMC started for Mothers
Q11.iii.KMCWherefromMotherTH1CHC2PHC3Pvt4Home5DHRO6	Where KMC started from as reported by mother<br>1= TH, 2=CHC, 3=PHC, 4=Pvt, 5=Home
WHO_KMCD1hours	KMC day 1 hours
DAY_KMC_Initiated	Day when KMC was initiated
WHO_KMClt24hours	KMC duration 24 hours before discharge
days_KMC3_RR	KMC initiated category >3 days, <=3 days
KMC24hours	<8 hours, >= 8 hours
KMC_7day	<8 hours, >= 8 hours
WHO_BFlt24Discharge	Breastfeeding before discharge, 1- Yes, 0 - No
M_KMCInitiatedFac1Home0	KMC initiated 0=Home, 1=Facility
KMC28	<8 hours, >= 8 hours
WHO_KMC_D7	KMC duration 7th day hours
WHO_EBF_D7	Exclusive Breastfeeding on 7th day
WHO_KMC_D28hours	KMC duration on 28th day - hours
WHO_EBF_day28	Exclusive Breastfeeding 28 days
M_DateofInterview	Date of visit for completing questionnaire with mothers
M_Q13.i.KMCendeddateifcontinuingDateofinterviewwasput	Date of continuing KMC
M_NoofDaysKMCgive	No of days KMC given
M_Q16.FollowuptohospitalY1N0	Follow-up hospital 1=Yes, 0=No

M_Qs22.i.DInterviewEBFY1Anyother0	Exclusive Breastfeeding 1=Yes, 0=Others
M_Q6.HealthStatusDayofInterviewWell1Sick0	1=Well, 0=Sick
Q9.Durationdayscolouredifnormalstay	Number of days admitted in the hospital
Hospcat	Hospital stay category
Q17.M_Ageyrs	Age of the mothers
Q18.MEducationYears	Education of the mothers
Meducat	Education of the mothers - category
M_Occ_Skilled1Unskilled2Business3Homemaker4	Occupation of the mothers<br>1=Skilled, 2=Unskilled, 3=Business,<br>4=Homemaker
Q19.MOccupation1.Skilled2unskilled3business4HW	Occupation of the mothers
Q21.ii.M_AgeofSpouseyrs	Age of the spouse
Q20.M_EducationSpouse	Education of the spouse
Q21.i.M_OccSpouse	Occupation of the spouse
M_SpouseOcc	Occupation of the spouse category
Q24.i.M_NoofChildren	Number of children
M_NoChildrenPrimi112gt23	1=Primi, 2=others
M_Knowledge_TOTAL30	Knowledge of the mothers
M_HelpedIniTotQ127Y10HCP122344566	Who helped mother in KMC initiation<br>0=None, 1=Yes+,<br>2=1-2 persons, 3= 3-4 persons, 4= >4 persons
M_CounselledTOTQ137BA10if122if344if566	Who counselled the mother for KMC initiation<br>0=None, 1 = Yes+<br>1 for each open-response provided
M_SupIniKMC_14	The score for KMC initiation support
M_HelpedMostTotQ14BI1or000HCP12234354	Who helped mother for KMC maintenance in facility<br>0=None, 1 = Yes+,<br>2=1- 2 persons, 3= 3-4 persons, 4= >4 persons
M_Q15.KMCKitY5N0	KMC kit 5=Yes, 0=No
FKMC_HospQ18.Y5No0Grey_noFKMC	Availability of fKMC provider<br>0=No, 5=Yes
M_SupMaintenanceHospital_Tot15	Total score for KMC maintenance support in the<br>facility
M_SupportFac_29	Overall KMC support at facility
M_ASHAHelpQ17_6	Help received from ASHA for KMC maintenance at<br>home
M_Q18TotSupHome_2for1person	Number of persons giving most support at home
M_SuppHomeHelpedMostScore_0Nil3gt1	Number of persons giving most support at home<br>0=No support, 3= 1 or more persons
M_NoofFMtohelp_Actual	Number of family members helping mothers.
NoFMHelpedScore001122gt33	Number of family members helping mothers.<br>0=no support, 1=1 person, 2=2 persons

	3= >2 persons
FKMCProvideratHomeY5N0	fKMC provider available at home<br>5=Yes, 0= No
FKMC_TOT_K30	Knowledge score of fKMC provider
FKMC_TOT_SUPPORT18	Support score of fKMC provider
FKMC_TOT_Att4	Attitude score of fKMC provider
MorFKMCreported_Q5.KMCdurationdayhrs	Hours of KMC provided by fKMC provider
M_SupHomeTOTAL38	Total support at home
