# Supplementary material for: An exploration of the support received by mothers for kangaroo mother care practice along the health facility-community continuum in a sub-district of Northern Karnataka, India
Source: PLoS One. 2025 Mar 6;20(3):e0308738. doi: 10.1371/journal.pone.0308738 (PMC11884690; doi:10.1371/journal.pone.0308738)
Supplement: S2 Data — (PDF) [file pone.0308738.s003.pdf]

PID	S.No	rthWeightG	BWcat	5.SexM1F2(	TH1CHC2PH	placebirth	HC2PHC3P\	Admitted
1	1	2000	>1500	2	5	Homes	1	Public
2	1	1750	>1500	2	1	Public	1	Public
3	2	1800	>1500	2	3	Public	1	Public
4	3	1550	>1500	2	3	Public	3	Public
5	4	1860	>1500	1	2	Public	4	Private
6	5	1900	>1500	2	2	Public	2	Public
7	6	1650	>1500	1	4	Private	4	Private
8	7	1600	>1500	1	3	Public	3	Public
9	8	1600	>1500	2	2	Public	4	Private
10	8	1750	>1500	2	2	Public	4	Private
11	9	1655	>1500	1	4	Private	4	Private
12	10	1980	>1500	2	3	Public	3	Public
13	11	1800	>1500	2	1	Public	4	Private
14	12	1620	>1500	1	3	Public	1	Public
15	13	1900	>1500	1	5	Homes	3	Public
16	14	1600	>1500	1	1	Public	4	Private
17	15	1600	>1500	2	1	Public	4	Private
18	16	1420	<=1500	2	4	Private	4	Private
19	17	1610	>1500	2	4	Private	4	Private
20	38	1600	>1500	2	4	Private	4	Private
21	18	1900	>1500	2	1	Public	4	Private
22	19	1900	>1500	1	1	Public	1	Public
23	20	1870	>1500	1	5	Homes	3	Public
24	21	1900	>1500	2	1	Public	1	Public
25	22	1700	>1500	2	2	Public	2	Public
26	23	1600	>1500	1	1	Public	1	Public
27	24	1880	>1500	2	2	Public	2	Public
28	25	1540	>1500	1	5	Homes	4	Private
29	26	1900	>1500	1	1	Public	4	Private
30	27	1200	<=1500	1	1	Public	4	Private
31	28	1980	>1500	1	3	Public	1	Public
32	29	1600	>1500	1	1	Public	1	Public
33	30	1400	<=1500	1	1	Public	4	Private
34	31	1620	>1500	1	3	Public	1	Public
35	32	1878	>1500	1	3	Public	1	Public
36	33	1900	>1500	2	4	Private	4	Private
37	34	1700	>1500	2	3	Public	3	Public
38	35	1400	<=1500	2	3	Public	4	Private
39	36	1900	>1500	2	3	Public	4	Private
40	37	1900	>1500	2	4	Private	4	Private
41	39	1700	>1500	1	5	Homes	3	Public
42	40	1740	>1500	2	2	Public	2	Public
43	41	1700	>1500	2	4	Private	4	Private
44	42	1735	>1500	1	4	Private	4	Private
45	43	1500	<=1500	2	4	Private	4	Private
46	44	1800	>1500	1	1	Public	1	Public

47	45	1370	<=1500	2	4	Private	4	Private
48	45	1460	<=1500	2	4	Private	4	Private
49	46	1950	>1500	1	4	Private	4	Private
50	47	1600	>1500	1	4	Private	4	Private
51	48	1800	>1500	1	1	Public	4	Private
52	49	1720	>1500	2	3	Public	3	Public
53	50	1075	<=1500	2	4	Private	4	Private
54	51	1780	>1500	2	4	Private	4	Private
55	52	1790	>1500	2	3	Public	3	Public
56	53	1700	>1500	1	1	Public	4	Private
57	54	1800	>1500	1	1	Public	4	Private
58	55	1740	>1500	1	3	Public	3	Public
59	56	1890	>1500	2	3	Public	3	Public
60	57	1900	>1500	2	1	Public	1	Public
61	58	1800	>1500	1	5	Homes	2	Public
62	59	1500	<=1500	2	4	Private	4	Private
63	60	1960	>1500	2	2	Public	2	Public
64	61	1900	>1500	1	1	Public	4	Private
65	62	1900	>1500	1	1	Public	1	Public
66	63	1700	>1500	2	1	Public	1	Public
67	64	1365	<=1500	2	4	Private	4	Private
68	65	1800	>1500	1	3	Public	4	Private
69	66	1635	>1500	2	3	Public	4	Private
70	67	1200	<=1500	1	3	Public	4	Private
71	68	1820	>1500	2	3	Public	3	Public
72	69	1900	>1500	2	1	Public	4	Private
73	70	1900	>1500	2	3	Public	3	Public
74	71	1630	>1500	1	5	Homes	3	Public
75	72	1900	>1500	2	1	Public	4	Private
76	73	1250	<=1500	1	4	Private	4	Private
77	73	1520	>1500	2	4	Private	4	Private
78	74	1700	>1500	2	1	Public	1	Public
79	75	1600	>1500	2	1	Public	4	Private
80	76	1840	>1500	1	3	Public	3	Public
81	77	1800	>1500	2	2	Public	4	Private
82	78	1900	>1500	1	1	Public	1	Public
83	79	1560	>1500	1	4	Private	4	Private
84	80	1810	>1500	1	3	Public	3	Public
85	81	1600	>1500	2	1	Public	1	Public
86	82	1800	>1500	2	1	Public	4	Private
87	83	1850	>1500	2	1	Public	1	Public
88	84	1500	<=1500	1	1	Public	4	Private
89	85	1750	>1500	2	4	Private	4	Private
90	86	1200	<=1500	2	1	Public	4	Private
91	87	1900	>1500	2	1	Public	4	Private
92	88	1990	>1500	1	4	Private	4	Private
93	89	1770	>1500	2	4	Private	4	Private

94	90	1800	>1500	1	1	Public	1	Public
95	91	1800	>1500	1	1	Public	1	Public
96	92	1930	>1500	1	4	Private	4	Private
97	92	1830	>1500	1	4	Private	4	Private
98	93	1750	>1500	2	5	Homes	3	Public
99	94	1900	>1500	1	1	Public	1	Public
100	95	1740	>1500	2	2	Public	4	Private
101	96	1500	<=1500	2	1	Public	1	Public
102	97	1660	>1500	2	1	Public	4	Private
103	98	1800	>1500	2	1	Public	1	Public
104	99	1200	<=1500	2	4	Private	4	Private
105	99	1500	<=1500	1	4	Private	4	Private
106	100	1700	>1500	2	4	Private	4	Private
107	100	1700	>1500	2	4	Private	4	Private
108	101	1800	>1500	2	1	Public	1	Public
109	102	1700	>1500	2	3	Public	3	Public
110	103	1700	>1500	2	3	Public	4	Private
111	104	1900	>1500	2	2	Public	2	Public
112	105	1200	<=1500	2	2	Public	4	Private
113	106	1800	>1500	2	2	Public	2	Public
114	107	1700	>1500	2	4	Private	4	Private
115	108	1800	>1500	1	4	Private	4	Private
116	109	1970	>1500	1	2	Public	2	Public
117	110	1800	>1500	2	1	Public	4	Private
118	111	1700	>1500	2	1	Public	1	Public
119	112	1700	>1500	2	4	Private	4	Private
120	113	1650	>1500	1	4	Private	4	Private
121	113	1716	>1500	1	4	Private	4	Private
122	114	1500	<=1500	1	1	Public	1	Public
123	114	1200	<=1500	2	1	Public	1	Public
124	115	1500	<=1500	1	4	Private	4	Private
125	116	1600	>1500	2	1	Public	1	Public
126	117	1200	<=1500	2	1	Public	4	Private
127	118	1270	<=1500	2	4	Private	4	Private
128	119	1500	<=1500	2	4	Private	4	Private
129	119	1565	>1500	1	4	Private	4	Private
130	120	1800	>1500	2	1	Public	1	Public
131	121	1915	>1500	2	4	Private	4	Private
132	122	1930	>1500	1	4	Private	4	Private
133	123	1800	>1500	1	1	Public	1	Public
134	124	1600	>1500	1	1	Public	1	Public
135	125	1600	>1500	1	1	Public	1	Public
136	126	1850	>1500	2	4	Private	1	Public
137	127	1980	>1500	1	4	Private	4	Private
138	128	1940	>1500	1	5	Homes	2	Public
139	129	1780	>1500	2	5	Homes	2	Public
140	130	1980	>1500	2	1	Public	1	Public

141	131	1900	>1500	2	1	Public	1	Public
142	132	1770	>1500	2	4	Private	4	Private
143	133	1520	>1500	1	2	Public	2	Public
144	134	1600	>1500	1	1	Public	1	Public
145	135	1660	>1500	2	1	Public	1	Public
146	136	1880	>1500	2	4	Private	4	Private
147	137	1800	>1500	1	1	Public	1	Public
148	138	1280	<=1500	2	4	Private	4	Private
149	139	1880	>1500	2	1	Public	1	Public
150	140	1750	>1500	2	1	Public	1	Public
151	141	1800	>1500	2	1	Public	1	Public
152	142	1250	<=1500	2	5	Homes	1	Public
153	143	1130	<=1500	2	4	Private	1	Public
154	144	1200	<=1500	1	5	Homes	4	Private
155	145	1620	>1500	1	4	Private	4	Private
156	145	1380	<=1500	1	4	Private	4	Private
157	146	1800	>1500	1	4	Private	4	Private
158	147	1800	>1500	2	5	Homes	2	Public
159	148	1500	<=1500	2	1	Public	4	Private
160	148	1300	<=1500	2	1	Public	4	Private
161	149	1600	>1500	1	1	Public	3	Public
162	150	1900	>1500	1	4	Private	4	Private
163	151	1500	<=1500	1	4	Private	4	Private
164	151	2000	>1500	1	4	Private	4	Private
165	152	1600	>1500	2	1	Public	1	Public
166	153	1900	>1500	2	1	Public	1	Public
167	154	1900	>1500	2	1	Public	1	Public
168	155	1900	>1500	1	1	Public	4	Private
169	156	1600	>1500	2	4	Private	4	Private
170	157	1200	<=1500	2	4	Private	4	Private
171	157	1000	<=1500	2	4	Private	4	Private
172	158	1500	<=1500	1	5	Homes	4	Private
173	159	1970	>1500	2	5	Homes	2	Public
174	160	1800	>1500	2	1	Public	1	Public
175	161	1900	>1500	2	1	Public	1	Public
176	162	1790	>1500	2	3	Public	1	Public
177	163	1130	<=1500	2	4	Private	1	Public
178	164	1600	>1500	2	4	Private	4	Private
179	165	1990	>1500	1	4	Private	4	Private
180	166	1250	<=1500	2	2	Public	1	Public
181	167	1900	>1500	2	5	Homes	1	Public
182	168	1900	>1500	1	2	Public	4	Private
183	169	1700	>1500	1	1	Public	4	Private
184	169	1500	<=1500	2	1	Public	4	Private
185	170	1500	<=1500	1	1	Public	4	Private
186	171	1720	>1500	1	4	Private	4	Private
187	172	1400	<=1500	1	4	Private	4	Private

188	173	1800	>1500	2	1	Public	1	Public
189	174	1800	>1500	2	4	Private	4	Private
190	175	1980	>1500	1	2	Public	2	Public
191	176	1661	>1500	1	3	Public	4	Private
192	177	1900	>1500	2	5	Homes	1	Public
193	178	1700	>1500	2	5	Homes	3	Public
194	179	1850	>1500	1	4	Private	4	Private
195	180	2000	>1500	2	4	Private	4	Private
196	181	1950	>1500	2	1	Public	1	Public
197	182	1640	>1500	2	4	Private	4	Private
198	183	1900	>1500	2	1	Public	1	Public
199	184	1900	>1500	2	4	Private	4	Private
200	185	1960	>1500	1	2	Public	1	Public
201	186	1500	<=1500	1	4	Private	4	Private
202	187	1500	<=1500	2	5	Homes	1	Public
203	187	1500	<=1500	2	5	Homes	1	Public
204	188	1700	>1500	2	1	Public	1	Public
205	189	1500	<=1500	2	4	Private	4	Private
206	190	1400	<=1500	2	1	Public	1	Public
207	191	1800	>1500	2	4	Private	4	Private
208	192	1770	>1500	2	3	Public	4	Private
209	193	1900	>1500	2	4	Private	4	Private
210	194	1660	>1500	1	2	Public	4	Private
211	195	1400	<=1500	1	4	Private	4	Private
212	196	1900	>1500	1	4	Private	4	Private
213	197	1200	<=1500	1	4	Private	4	Private
214	198	1300	<=1500	1	1	Public	1	Public
215	199	1759	>1500	2	3	Public	1	Public
216	200	1910	>1500	2	4	Private	4	Private
217	201	1900	>1500	2	4	Private	4	Private
218	202	1960	>1500	2	1	Public	1	Public
219	203	1760	>1500	1	3	Public	3	Public
220	204	1900	>1500	2	1	Public	1	Public
221	205	1800	>1500	1	4	Private	4	Private
222	205	1880	>1500	2	4	Private	4	Private
223	206	1500	<=1500	1	2	Public	4	Private
224	207	1900	>1500	1	1	Public	1	Public
225	208	1900	>1500	2	1	Public	1	Public
226	208	1800	>1500	1	1	Public	1	Public
227	209	1560	>1500	1	4	Private	4	Private

2Well1Sick2	Q1.DOB	.ii.DateKMCstarted	therTH1CH	_KMCD1h	_KMC_Initi	_KMClt24h	ys_KMC3_	KMC24hour
1	22-Dec-17	23-Dec-17	1	8.30	2.00	2.30	<=3 days	<=8 hours
2	22-Dec-17	23-Dec-17	1	8.30	2.00	2.30	<=3 days	<=8 hours
2	9-Dec-17	16-Dec-17	3	9.00	8.00	17.00	>3 days	>8 hours
2	15-Dec-17	15-Dec-17	3	3.00	1.00	7.00	<=3 days	<=8 hours
2	26-Dec-17	11-Jan-18	4	6.00	17.00	9.30	>3 days	>8 hours
2	26-Dec-17	26-Dec-17	2	0.00	1.00	0.00	<=3 days	<=8 hours
2	20-Dec-17	25-Dec-17	4	8.00	6.00	8.30	>3 days	>8 hours
2	10-Dec-17	10-Dec-17	3	3.30	1.00	7.30	<=3 days	<=8 hours
2	17-Dec-17	19-Dec-17	2	3.30	3.00	3.30	<=3 days	<=8 hours
2	17-Dec-17	19-Dec-17	2	3.30	3.00	3.30	<=3 days	<=8 hours
2	26-Dec-17	31-Dec-17	4	8.00	6.00	0.00	>3 days	<=8 hours
2	3-Jan-18	3-Jan-18	3	6.00	1.00	6.00	<=3 days	<=8 hours
2	10-Jan-18	13-Jan-18	4	6.00	4.00	9.00	>3 days	>8 hours
2	14-Jan-18	15-Jan-18	3	12.50	2.00	11.00	<=3 days	>8 hours
2	3-Jan-18	4-Jan-18	3	4.00	2.00	4.00	<=3 days	<=8 hours
2	30-Dec-17	1-Jan-18	4	0.00	3.00	0.00	<=3 days	<=8 hours
2	9-Jan-18			0.00		0.00		<=8 hours
2	27-Dec-17	31-Dec-17	4	8.00	5.00	8.00	>3 days	>8 hours
1	30-Dec-17	4-Jan-18	5		6.00		>3 days	
1	9-Jan-18	11-Feb-18	5	2.45	34.00	11.45	>3 days	>8 hours
2	18-Jan-18	25-Jan-18	5	8.00	8.00	11.00	>3 days	>8 hours
2	19-Jan-18	19-Jan-18	1	3.30	1.00	11.30	<=3 days	>8 hours
2	16-Jan-18	16-Jan-18	3	2.00	1.00	2.00	<=3 days	<=8 hours
2	17-Jan-18	18-Jan-18	1	14.00	2.00	14.00	<=3 days	>8 hours
2	19-Jan-18	20-Jan-18	2	7.00	2.00	5.00	<=3 days	<=8 hours
2	17-Jan-18	19-Jan-18	4	4.00	3.00	4.00	<=3 days	<=8 hours
2	16-Jan-18	16-Jan-18	2	0.00	1.00	0.00	<=3 days	<=8 hours
2	28-Jan-18	30-Jan-18	4	3.00	3.00	3.00	<=3 days	<=8 hours
2	28-Jan-18	4-Feb-18	4	2.00	8.00	9.00	>3 days	>8 hours
2	2-Feb-18	8-Feb-18	4	16.00	7.00	16.00	>3 days	>8 hours
2	1-Feb-18	3-Feb-18	5	1.00	3.00	1.00	<=3 days	<=8 hours
2	7-Feb-18	7-Feb-18	1	1.00	1.00	12.00	<=3 days	>8 hours
2	28-Jan-18	3-Feb-18	4	1.30	7.00	0.00	>3 days	<=8 hours
2	27-Jan-18	27-Jan-18	3	15.20	1.00	12.30	<=3 days	>8 hours
1	27-Jan-18	29-Jan-18	3	10.30	3.00	10.30	<=3 days	>8 hours
1	30-Jan-18	5-Feb-18	5	3.00	7.00	4.00	>3 days	<=8 hours
2	3-Feb-18	3-Feb-18	3	8.10	1.00	8.30	<=3 days	>8 hours
2	27-Jan-18	29-Jan-18	3	5.00	3.00	5.00	<=3 days	<=8 hours
2	5-Feb-18	5-Feb-18	3	0.00	1.00	0.00	<=3 days	<=8 hours
2	31-Jan-18	1-Feb-18	5		2.00		<=3 days	
2	11-Feb-18	11-Feb-18	3	8.30	1.00	3.30	<=3 days	<=8 hours
2	20-Feb-18	20-Feb-18	2	9.30	1.00	9.00	<=3 days	>8 hours
1	30-Jan-18	8-Feb-18	4	2.30	10.00	2.30	>3 days	<=8 hours
1	5-Feb-18	6-Feb-18	4	3.00	2.00	5.00	<=3 days	<=8 hours
1	7-Feb-18	12-Feb-18	4	2.00	6.00	0.00	>3 days	<=8 hours
2	21-Feb-18	22-Feb-18	1	9.30	2.00	9.30	<=3 days	>8 hours

2	24-Feb-18	24-Feb-18	4	0.00	1.00	0.00	<=3 days	<=8 hours
1	24-Feb-18	24-Feb-18	4	0.00	1.00	0.00	<=3 days	<=8 hours
2	6-Mar-18	9-Mar-18	4	1.30	4.00	1.30	>3 days	<=8 hours
2	20-Feb-18	26-Feb-18	4	8.00	7.00	5.00	>3 days	<=8 hours
2	6-Mar-18	10-Mar-18	4	1.30	5.00	5.30	>3 days	<=8 hours
2	8-Mar-18	8-Mar-18	3	8.30	1.00	14.00	<=3 days	>8 hours
1	22-Mar-18	23-Mar-18	4	0.00	2.00	5.00	<=3 days	<=8 hours
1	9-Mar-18	14-Mar-18	4	1.30	6.00	5.00	>3 days	<=8 hours
2	12-Mar-18	12-Mar-18	3	8.00	1.00	10.00	<=3 days	>8 hours
2	1-Mar-18	1-Mar-18	1	4.00	1.00	7.30	<=3 days	<=8 hours
2	6-Mar-18	10-Mar-18	4	10.00	5.00	5.00	>3 days	<=8 hours
2	10-Mar-18	10-Mar-18	3	6.00	1.00	6.00	<=3 days	<=8 hours
2	27-Feb-18	27-Feb-18	3	2.20	1.00	2.00	<=3 days	<=8 hours
2	26-Feb-18	26-Feb-18	1	9.45	1.00	4.10	<=3 days	<=8 hours
2	27-Feb-18	27-Feb-18	3	2.20	1.00	2.20	<=3 days	<=8 hours
2	3-Mar-18	5-Mar-18	4	2.00	3.00	4.00	<=3 days	<=8 hours
2	12-Mar-18	12-Mar-18	2	5.00	1.00	13.00	<=3 days	>8 hours
2	11-Apr-18	11-Apr-18	1	6.00	1.00	5.30	<=3 days	<=8 hours
2	13-Apr-18	14-Apr-18	1	0.00	2.00	9.00	<=3 days	>8 hours
2	2-Apr-18	2-Apr-18	1	9.00	1.00	9.00	<=3 days	>8 hours
1	29-Mar-18	10-Apr-18	4	1.30	13.00	11.00	>3 days	>8 hours
2	28-Mar-18	29-Mar-18	4	13.00	2.00	7.30	<=3 days	<=8 hours
2	3-Apr-18	6-Apr-18	4	4.00	4.00	0.00	>3 days	<=8 hours
2	2-Apr-18	6-Apr-18	4	5.00	5.00	0.00	>3 days	<=8 hours
2	23-Mar-18	23-Mar-18	3	2.00	1.00	2.00	<=3 days	<=8 hours
2	5-Apr-18	7-Apr-18	4	12.00	3.00	4.00	<=3 days	<=8 hours
2	31-Mar-18	31-Mar-18	3	0.00	1.00	12.30	<=3 days	>8 hours
2	12-Apr-18	17-Apr-18	5	2.00	6.00	9.00	>3 days	>8 hours
2	12-Apr-18	13-Apr-18	1	0.00	2.00	7.00	<=3 days	<=8 hours
1	5-Apr-18		4	6.00		15.00		>8 hours
1	5-Apr-18		4	0.00		14.00		>8 hours
2	28-Mar-18	28-Mar-18	1	0.00	1.00	0.00	<=3 days	<=8 hours
2	20-Apr-18	20-Apr-18	1	5.00	1.00	13.00	<=3 days	>8 hours
2	22-Apr-18	23-Apr-18	3	10.00	2.00	10.00	<=3 days	>8 hours
2	10-May-18	17-May-18	5	8.50	8.00	0.00	>3 days	<=8 hours
2	14-May-18	15-May-18	1	20.00	2.00	8.00	<=3 days	>8 hours
1	12-May-18	21-May-18	5	4.00	10.00	2.00	>3 days	<=8 hours
2	26-Apr-18	26-Apr-18	3	0.00	1.00	11.00	<=3 days	>8 hours
2	6-May-18	6-May-18	1	10.00	1.00	12.00	<=3 days	>8 hours
2	23-Apr-18	26-Apr-18	4	5.00	4.00	0.00	>3 days	<=8 hours
2	27-Apr-18	28-Apr-18	1	8.00	2.00	11.00	<=3 days	>8 hours
2	24-Apr-18		4	5.10		14.50		>8 hours
1	3-May-18	10-May-18	5	6.00	8.00	2.00	>3 days	<=8 hours
2	4-May-18	5-May-18	4	5.00	2.00	0.00	<=3 days	<=8 hours
2	26-Apr-18	27-Apr-18	1	11.00	2.00	7.00	<=3 days	<=8 hours
1	27-Apr-18	1-May-18	4		5.00		>3 days	
1	23-Apr-18	26-Apr-18	5		4.00		>3 days	

2	4-May-18	4-May-18	1	18.30	1.00	8.30	<=3 days	>8 hours
2	17-Apr-18	20-Apr-18	1	0.00	4.00	13.50	>3 days	>8 hours
2	30-Apr-18	6-May-18	4	6.00	7.00	8.00	>3 days	>8 hours
2	30-Apr-18	6-May-18	4	6.00	7.00	8.00	>3 days	>8 hours
2	23-Apr-18	23-Apr-18	3	0.00	1.00	8.30	<=3 days	>8 hours
2	3-May-18	11-May-18	5	2.00	9.00	10.00	>3 days	>8 hours
2	7-May-18	10-May-18	5	7.00	4.00	1.00	>3 days	<=8 hours
2	9-Jun-18	11-Jun-18	1	2.00	3.00	0.00	<=3 days	<=8 hours
2	3-Jun-18	8-Jun-18	4	6.00	6.00	6.00	>3 days	<=8 hours
2	1-Jun-18	2-Jun-18	1	13.00	2.00	10.00	<=3 days	>8 hours
2	26-May-18	1-Jun-18	4	4.00	7.00	6.00	>3 days	<=8 hours
2	26-May-18	1-Jun-18	4	6.00	7.00	2.00	>3 days	<=8 hours
1	2-Jun-18	5-Jun-18	4	5.00	4.00	3.00	>3 days	<=8 hours
1	2-Jun-18	5-Jun-18	4	5.00	4.00	3.00	>3 days	<=8 hours
2	6-Jun-18	7-Jun-18	1	0.00	2.00	7.30	<=3 days	<=8 hours
1	9-Jun-18	9-Jun-18	3	0.00	1.00	21.00	<=3 days	>8 hours
2	13-May-18	15-May-18	4		3.00		<=3 days	
2	22-May-18	22-May-18	2	0.00	1.00	4.00	<=3 days	<=8 hours
2	31-May-18	13-Jun-18	5		14.00		>3 days	
2	1-Jun-18	2-Jun-18	2	4.00	2.00	9.00	<=3 days	>8 hours
2	16-May-18	22-Jun-18	5		38.00		>3 days	
1	12-Jun-18	13-Jun-18	4	4.30	2.00	1.30	<=3 days	<=8 hours
2	13-Jun-18	13-Jun-18	2	2.00	1.00	8.00	<=3 days	>8 hours
2	2-Jun-18	3-Jun-18	4	8.00	2.00	4.30	<=3 days	<=8 hours
2	2-Apr-18	3-Apr-18	1	9.00	2.00	11.00	<=3 days	>8 hours
1	31-May-18	1-Jun-18	4	2.00	2.00	0.00	<=3 days	<=8 hours
2	20-Jun-18	24-Jun-18	4	7.00	5.00	8.00	>3 days	>8 hours
2	20-Jun-18	24-Jun-18	4	8.50	5.00	9.00	>3 days	>8 hours
2	1-Jun-18	1-Jun-18	1	9.00	1.00	7.00	<=3 days	<=8 hours
2	1-Jun-18	1-Jun-18	1	8.00	1.00	11.00	<=3 days	>8 hours
1	21-May-18	16-Jun-18	5	3.00	27.00	9.00	>3 days	>8 hours
2	21-Jun-18	21-Jun-18	1	0.00	1.00	12.00	<=3 days	>8 hours
2	1-Jul-18	6-Jul-18	4	8.00	6.00	4.00	>3 days	<=8 hours
2	26-Jun-18	5-Jul-18	4	10.00	10.00	2.00	>3 days	<=8 hours
2	20-Jun-18	23-Jun-18	4	6.50	4.00	13.00	>3 days	>8 hours
2	20-Jun-18	23-Jun-18	4	8.50	4.00	0.00	>3 days	<=8 hours
2	18-Jul-18	19-Jul-18	1	13.00	2.00	10.30	<=3 days	>8 hours
2	19-May-18	23-May-18	4	7.30	5.00	5.00	>3 days	<=8 hours
1	27-Jun-18	1-Jul-18	4	4.00	5.00	8.30	>3 days	>8 hours
2	12-Jul-18	12-Jul-18	1	11.30	1.00	11.30	<=3 days	>8 hours
2	3-Jul-18	4-Jul-18	1	13.00	2.00	11.00	<=3 days	>8 hours
1	8-Jul-18	9-Jul-18	1	8.00	2.00	14.00	<=3 days	>8 hours
2	3-Jul-18	14-Jul-18	1	12.30	12.00	15.00	>3 days	>8 hours
2	29-Jun-18	1-Jul-18	4	7.00	3.00	9.00	<=3 days	>8 hours
2	4-Jul-18	4-Jul-18	2	7.00	1.00	11.30	<=3 days	>8 hours
2	1-Jul-18	1-Jul-18	2	8.00	1.00	11.20	<=3 days	>8 hours
2	14-Jun-18	14-Jun-18	1	0.00	1.00	9.00	<=3 days	>8 hours

2	30-Jun-18	1-Jul-18	1	8.00	2.00	9.30	<=3 days	>8 hours
2	2-Jul-18	9-Jul-18	4	0.00	8.00	8.00	>3 days	>8 hours
1	28-Jun-18	29-Jun-18	2	3.00	2.00	3.00	<=3 days	<=8 hours
2	10-Jul-18	12-Jul-18	1	11.00	3.00	10.30	<=3 days	>8 hours
2	22-Jun-18	26-Jun-18	1	8.00	5.00	15.20	>3 days	>8 hours
1	7-Jul-18	10-Jul-18	4	9.00	4.00	0.00	>3 days	<=8 hours
2	19-Jul-18	19-Jul-18	1	10.30	1.00	14.00	<=3 days	>8 hours
2	24-Jun-18	29-Jul-18	4	7.00	36.00	7.00	>3 days	<=8 hours
1	16-Jul-18	22-Jul-18	5	2.00	7.00	2.00	>3 days	<=8 hours
2	26-Jun-18	26-Jun-18	1	3.30	1.00	9.30	<=3 days	>8 hours
2	19-Jul-18	19-Jul-18	1	0.00	1.00	10.30	<=3 days	>8 hours
2	16-Jul-18	26-Jul-18	1	2.00	11.00	15.50	>3 days	>8 hours
2	26-Jul-18	26-Jul-18	4	4.00	1.00	18.00	<=3 days	>8 hours
2	29-Jun-18	6-Jul-18	4	12.00	8.00	17.30	>3 days	>8 hours
2	23-Jul-18	28-Jul-18	4	9.00	6.00	15.00	>3 days	>8 hours
2	23-Jul-18	27-Jul-18	4	9.00	5.00	11.30	>3 days	>8 hours
2	4-Aug-18	6-Aug-18	4	13.00	3.00	14.00	<=3 days	>8 hours
2	21-Jul-18	23-Jul-18	2	13.00	3.00	10.00	<=3 days	>8 hours
2	31-Jul-18	4-Aug-18	1	15.50	5.00	0.00	>3 days	<=8 hours
2	31-Jul-18	4-Aug-18	1	0.00	5.00	0.00	>3 days	<=8 hours
2	21-Jul-18	23-Jul-18	1	13.00	3.00	9.00	<=3 days	>8 hours
1	26-Jul-18	27-Jul-18	4	0.00	2.00	5.00	<=3 days	<=8 hours
2	3-Aug-18	3-Aug-18	4	2.00	1.00	8.00	<=3 days	>8 hours
1	3-Aug-18	3-Aug-18	4	5.00	1.00	8.00	<=3 days	>8 hours
2	31-Jul-18	31-Jul-18	1	9.00	1.00	10.00	<=3 days	>8 hours
2	4-Aug-18	4-Aug-18	1	8.30	1.00	11.00	<=3 days	>8 hours
2	21-Jul-18	4-Aug-18	5		15.00		>3 days	
2	27-Jul-18	29-Jul-18	4	15.20	3.00	11.00	<=3 days	>8 hours
2	5-Aug-18	7-Aug-18	4	0.00	3.00	18.00	<=3 days	>8 hours
2	28-Jul-18	3-Aug-18	4	5.00	7.00	8.00	>3 days	>8 hours
2	28-Jul-18	3-Aug-18	4	2.00	7.00	8.00	>3 days	>8 hours
2	7-Aug-18	9-Aug-18	4	7.00	3.00	8.00	<=3 days	>8 hours
2	30-Jul-18	30-Jul-18	4	0.00	1.00	13.30	<=3 days	>8 hours
2	8-Aug-18	9-Aug-18	1	0.00	2.00	10.00	<=3 days	>8 hours
2	3-Aug-18	4-Aug-18	1	14.30	2.00	11.00	<=3 days	>8 hours
2	2-Aug-18	15-Aug-18	1	15.00	14.00	18.00	>3 days	>8 hours
2	18-Jul-18	1-Aug-18	4	4.50	15.00	3.00	>3 days	<=8 hours
2	11-Aug-18	13-Aug-18	4	10.00	3.00	14.00	<=3 days	>8 hours
2	21-Aug-18	24-Aug-18	4	5.00	4.00	16.30	>3 days	>8 hours
2	19-Aug-18	19-Aug-18	3	0.00	1.00	18.10	<=3 days	>8 hours
2	23-Aug-18	24-Aug-18	1	10.00	2.00	10.00	<=3 days	>8 hours
2	24-Aug-18	28-Aug-18	4	12.00	5.00	8.00	>3 days	>8 hours
2	25-Aug-18	1-Sep-18	4	6.00	8.00	13.00	>3 days	>8 hours
2	25-Aug-18	1-Sep-18	4	4.00	8.00	13.00	>3 days	>8 hours
2	15-Aug-18	16-Aug-18	4	0.00	2.00	2.00	<=3 days	<=8 hours
2	4-Sep-18	9-Sep-18	4	8.00	6.00	6.30	>3 days	<=8 hours
2	5-Sep-18	9-Sep-18	4	3.00	5.00	12.00	>3 days	>8 hours

2	1-Sep-18	2-Sep-18	1	10.20	2.00	8.00	<=3 days	>8 hours
2	7-Sep-18	11-Sep-18	4	11.90	5.00	10.00	>3 days	>8 hours
2	30-Aug-18	30-Aug-18	2	0.00	1.00	12.00	<=3 days	>8 hours
2	6-Sep-18	8-Sep-18	4	11.00	3.00	8.30	<=3 days	>8 hours
2	28-Aug-18	29-Aug-18	1	0.00	2.00	11.00	<=3 days	>8 hours
2	17-Aug-18	17-Aug-18	3	0.00	1.00	11.00	<=3 days	>8 hours
2	21-Aug-18	22-Aug-18	4	15.00	2.00	10.00	<=3 days	>8 hours
1	22-Aug-18	27-Aug-18	4	12.00	6.00	12.00	>3 days	>8 hours
2	4-Sep-18	5-Sep-18	1	14.00	2.00	12.30	<=3 days	>8 hours
2	17-Aug-18	24-Aug-18	5		8.00		>3 days	
2	31-Aug-18	31-Aug-18	1	6.00	1.00	7.00	<=3 days	<=8 hours
2	19-Aug-18	21-Aug-18	4	7.00	3.00	3.00	<=3 days	<=8 hours
2	31-Aug-18	1-Sep-18	2	10.20	2.00	8.00	<=3 days	>8 hours
1	11-Sep-18	13-Sep-18	4	0.00	3.00	4.00	<=3 days	<=8 hours
2	15-Sep-18	15-Sep-18	1	16.00	1.00	8.30	<=3 days	>8 hours
2	15-Sep-18	15-Sep-18	1	7.00	1.00	16.00	<=3 days	>8 hours
2	15-Sep-18	18-Sep-18	5	4.00	4.00	17.30	>3 days	>8 hours
2	8-Sep-18	12-Sep-18	4	8.30	5.00	0.00	>3 days	<=8 hours
2	22-Sep-18	24-Sep-18	1	4.00	3.00	4.00	<=3 days	<=8 hours
1	21-Sep-18	21-Sep-18	4	7.00	1.00	3.00	<=3 days	<=8 hours
2	25-Sep-18	28-Sep-18	4	11.50	4.00	18.00	>3 days	>8 hours
2	23-Sep-18	5-Oct-18	5		13.00		>3 days	
2	24-Sep-18	25-Sep-18	4	9.00	2.00	5.00	<=3 days	<=8 hours
2	7-Sep-18	14-Sep-18	4	8.00	8.00	14.00	>3 days	>8 hours
2	10-Sep-18	17-Sep-18	4	14.40	8.00	3.00	>3 days	<=8 hours
2	18-Sep-18	24-Sep-18	4	11.00	7.00	5.50	>3 days	<=8 hours
2	19-Sep-18	30-Sep-18	1	0.00	12.00	16.30	>3 days	>8 hours
2	11-Sep-18	11-Sep-18	1	4.00	1.00	8.00	<=3 days	>8 hours
1	26-Sep-18	11-Oct-18	4	2.00	16.00	2.00	>3 days	<=8 hours
2	20-Sep-18	25-Sep-18	5		6.00		>3 days	
2	13-Sep-18	13-Sep-18	1	0.00	1.00	9.60	<=3 days	>8 hours
2	24-Sep-18	24-Sep-18	3	2.20	1.00	13.00	<=3 days	>8 hours
2	20-Sep-18	21-Sep-18	1	18.00	2.00	9.00	<=3 days	>8 hours
1	20-Sep-18	20-Sep-18	4	7.00	1.00	13.30	<=3 days	>8 hours
1	20-Sep-18	20-Sep-18	4	4.30	1.00	13.30	<=3 days	>8 hours
2	22-Sep-18	26-Sep-18	5	5.00	5.00	0.00	>3 days	<=8 hours
2	20-Sep-18	24-Sep-18	1	14.30	5.00	11.00	>3 days	>8 hours
2	23-Sep-18	24-Sep-18	1	7.00	2.00	10.00	<=3 days	>8 hours
2	23-Sep-18	24-Sep-18	1	13.00	2.00	10.00	<=3 days	>8 hours
2	24-Sep-18	2-Oct-18	4	6.00	9.00	6.00	>3 days	<=8 hours

KMC_7day	_BFlt24Disc	InitiatedFac	KMC28	WHO_KMC_D	WHO_EBF_D	_KMC_D28	HO_EBF_day	DateofInterv
<=8 hours	1	1		2	1			23-Jan-18
<=8 hours	1	1		2	1			23-Jan-18
>8 hours	1	1		20	1			24-Jan-18
>8 hours	1	1		12	1			24-Jan-18
<=8 hours	1	1		6	1			24-Jan-18
>8 hours	1	1		8	1			24-Jan-18
<=8 hours	1	1		6	1			25-Jan-18
>8 hours	1	1		8	1			25-Jan-18
<=8 hours	1	1		4	1			25-Jan-18
<=8 hours	1	1		4	1			25-Jan-18
>8 hours	1	1		12	1			25-Jan-18
>8 hours	1	1		10	1			9-Feb-18
>8 hours	1	1		10	1			9-Feb-18
>8 hours	1	1		9	1			9-Feb-18
>8 hours	1	1		9	1			9-Feb-18
>8 hours	1	1		9	1			9-Feb-18
<=8 hours	0	1		5	1			9-Feb-18
>8 hours	1	1		15	1			10-Feb-18
>8 hours		0		12	1			10-Feb-18
>8 hours	1	1		8	1			10-Feb-18
<=8 hours	1	1		4	1			17-Feb-18
<=8 hours	1	1		0	1			17-Feb-18
>8 hours	1	1		14	1			19-Feb-18
<=8 hours	1	1		4	1			1-Mar-18
		1						24-Feb-18
>8 hours	1	1		10	1			1-Mar-18
<=8 hours	1	1		6	1			19-Feb-18
>8 hours	1	1		9	1			9-Mar-18
		1						9-Mar-18
>8 hours	1	1		8	1			8-Mar-18
>8 hours	1	1		9	1			9-Mar-18
>8 hours	1	1		8	1			9-Mar-18
>8 hours	1	1		14	1			9-Mar-18
>8 hours	1	1		12	1			9-Mar-18
>8 hours	1	1		14	1			9-Mar-18
>8 hours	1	1		8	1			8-Mar-18
>8 hours	1	1		14	1			8-Mar-18
>8 hours	1	1		12	1			8-Mar-18
>8 hours	1	1		10	1			8-Mar-18
		0						8-Mar-18
>8 hours	1	1		14	1			14-Mar-18
>8 hours	1	1		14	1			21-Mar-18
>8 hours	1	1		12	1			14-Mar-18
>8 hours	1	1		12	1			16-Mar-18
>8 hours	1	1		12	1			14-Mar-18
<=8 hours	1	1		6	1			11-Apr-18

>8 hours		1		12	1		11-Apr-18
>8 hours		1		12	1		11-Apr-18
		1					11-Apr-18
>8 hours	1	1		16	1		11-Apr-18
<=8 hours	1	1		6	1		11-Apr-18
>8 hours	1	1	<=8 hours	9	1	5	11-Apr-18
>8 hours	1	1	>8 hours	8	1	8	11-Apr-18
<=8 hours	1	1	<=8 hours	0	1	0	11-Apr-18
<=8 hours	1	1	<=8 hours	6	1	2	11-Apr-18
<=8 hours	1	1	<=8 hours	0		0	12-Apr-18
<=8 hours	1	1	<=8 hours	6	1	4	12-Apr-18
<=8 hours	1	1	<=8 hours	4	1	2	12-Apr-18
<=8 hours	1	1	<=8 hours	6	1	4	12-Apr-18
>8 hours	1	1		10	1		12-Apr-18
>8 hours	1	1		8	1		12-Apr-18
>8 hours	1	1	<=8 hours	13	1	0	12-Apr-18
<=8 hours	1	1	<=8 hours	4	1	2	23-Apr-18
<=8 hours	0	1	<=8 hours	0	0	0	8-May-18
<=8 hours	1	1	>8 hours	6	1	9	8-May-18
>8 hours	1	1	<=8 hours	10	1	2	8-May-18
>8 hours	1	1	>8 hours	11	1	8	8-May-18
>8 hours	1	1	<=8 hours	9	1	0	8-May-18
<=8 hours	1	1	<=8 hours	0	1	0	8-May-18
<=8 hours	1	1	<=8 hours	0	0	0	8-May-18
<=8 hours	1	1	<=8 hours	6	1	2	9-May-18
>8 hours	1	1	<=8 hours	8	1	0	9-May-18
<=8 hours	1	1	<=8 hours	4	1	0	9-May-18
<=8 hours	1	1	<=8 hours	6	1	3	9-May-18
<=8 hours	1	1	<=8 hours	7	1	7	9-May-18
<=8 hours	1	1	<=8 hours	4	1	4	9-May-18
<=8 hours	1	1	<=8 hours	4	1	4	9-May-18
<=8 hours	1	1	<=8 hours	0	1	0	########
>8 hours	1	1	>8 hours	11	1	18	########
>8 hours	1	1	>8 hours	8	1	10	########
<=8 hours	1	1	<=8 hours	6	0	0	7-Jun-18
<=8 hours	1	1	<=8 hours	6	1	4	8-Jun-18
<=8 hours	1	1	<=8 hours	4	1	6	7-Jun-18
<=8 hours	1	1	<=8 hours	2	1	2	8-Jun-18
<=8 hours	1	1	<=8 hours	6	1	0	8-Jun-18
<=8 hours	1	1	<=8 hours	0	1	0	7-Jun-18
<=8 hours	1	1	<=8 hours	6	1	0	7-Jun-18
<=8 hours	0	1	<=8 hours	0	0	0	7-Jun-18
<=8 hours	1	1	<=8 hours	6	1	6	7-Jun-18
<=8 hours	1	1	<=8 hours	0	1	0	7-Jun-18
<=8 hours	1	1	<=8 hours	4	1	0	7-Jun-18
<=8 hours	1	0	<=8 hours	0	1	0	8-Jun-18
<=8 hours	1	0		0	1		8-Jun-18

>8 hours	1	1	<=8 hours	8	1	2	7-Jun-18
>8 hours	1	1	<=8 hours	8.5	1	6	8-Jun-18
>8 hours	1	1	<=8 hours	8	1	5	8-Jun-18
>8 hours	1	1	<=8 hours	8	1	5	8-Jun-18
>8 hours	1	1	>8 hours	8	1	9	10-Jun-18
<=8 hours	1	1	<=8 hours	6	1	0	11-Jun-18
>8 hours	1	1	<=8 hours	11	1	4	17-Jun-18
<=8 hours	0	1	<=8 hours	0	0	0	5-Jul-18
>8 hours	1	1	>8 hours	10	1	8	5-Jul-18
<=8 hours	1	1	<=8 hours	4	1	0	5-Jul-18
<=8 hours	1	1	>8 hours	7	1	9.5	5-Jul-18
<=8 hours	1	1	>8 hours	7	1	9.5	5-Jul-18
>8 hours	1	1	<=8 hours	8	1	4	5-Jul-18
>8 hours	1	1	<=8 hours	8	1	4	5-Jul-18
<=8 hours	1	1	<=8 hours	0	1	0	5-Jul-18
>8 hours	1	1	>8 hours	8	1	8	5-Jul-18
		0	<=8 hours		0	0	6-Jul-18
<=8 hours	1	1	<=8 hours	3	1	4	6-Jul-18
<=8 hours	0	0	<=8 hours	0	0	0	6-Jul-18
<=8 hours	1	1	<=8 hours	6	1	7	6-Jul-18
		0			0		6-Jul-18
<=8 hours	1	1	<=8 hours	5	1	4	6-Jul-18
<=8 hours	1	1	<=8 hours	6	1	4	6-Jul-18
<=8 hours	1	1	<=8 hours	0	0	0	6-Jul-18
<=8 hours	1	1	<=8 hours	2	1	1	########
<=8 hours	1	1	<=8 hours	0	1	0	5-Jul-18
<=8 hours	1	1	<=8 hours	6	1	3	11-Jul-18
<=8 hours	1	1	<=8 hours	6	1	3	11-Jul-18
<=8 hours	1	1	>8 hours	7.5	1	8	16-Jul-18
>8 hours	1	1	<=8 hours	8	1	6	16-Jul-18
>8 hours	1	1	<=8 hours	11	1	4	17-Jul-18
<=8 hours	1	1	<=8 hours	3	1	4	18-Jul-18
<=8 hours	1	1	<=8 hours	7.5	1	5.5	21-Jul-18
>8 hours	1	1	<=8 hours	11	1	6	25-Jul-18
<=8 hours	1	1	<=8 hours	5	0	0	26-Jul-18
<=8 hours	1	1	<=8 hours	0	0	0	26-Jul-18
>8 hours	1	1		12	0		3-Aug-18
>8 hours	1	1	<=8 hours	8	1	6	7-Jul-18
>8 hours	1	1	>8 hours	9	1	9	8-Aug-18
>8 hours	1	1	<=8 hours	8	1	3	8-Aug-18
>8 hours	1	1	>8 hours	10	1	8	8-Aug-18
>8 hours	1	1	>8 hours	14	1	9	8-Aug-18
>8 hours	1	1	>8 hours	12	1	8	9-Aug-18
<=8 hours	1	1	<=8 hours	0	0	0	9-Aug-18
>8 hours	1	1	>8 hours	8	1	8	9-Aug-18
<=8 hours	1	1	>8 hours	3	1	8	9-Aug-18
<=8 hours	1	1	<=8 hours	2.5	1	7	9-Aug-18

<=8 hours	1	1	<=8 hours	3	1	3	9-Aug-18
<=8 hours	1	1	<=8 hours	0	0	0	9-Aug-18
<=8 hours	1	1	<=8 hours	6	1	0	9-Aug-18
>8 hours	1	1	<=8 hours	8	1	6.2	9-Aug-18
<=8 hours	1	1	<=8 hours	2.5	1	2.5	9-Aug-18
<=8 hours	1	1	<=8 hours	0	0	0	9-Aug-18
>8 hours	1	1	<=8 hours	8	1	0	10-Aug-18
>8 hours	1	1	>8 hours	12	1	10	10-Aug-18
<=8 hours	1	1	<=8 hours	2	1	6	10-Aug-18
<=8 hours	1	1	<=8 hours	0	1	0	21-Jul-18
>8 hours	1	1	<=8 hours	8.5	1	5.5	10-Aug-18
>8 hours	1	1	>8 hours	13	1	9	18-Aug-18
>8 hours	1	1	<=8 hours	10	1	6	19-Aug-18
>8 hours	1	1	<=8 hours	15	1	4	19-Aug-18
<=8 hours	0	1	<=8 hours	0	0	0	22-Aug-18
>8 hours	1	1	<=8 hours	10.8	0	0	22-Aug-18
<=8 hours	1	1	<=8 hours	0	1	0	31-Aug-18
>8 hours	1	1	>8 hours	10	1	8	22-Aug-18
<=8 hours	0	1	<=8 hours	0	0	0	31-Aug-18
<=8 hours	0	1	<=8 hours	0	0	0	31-Aug-18
<=8 hours	1	1	<=8 hours	3	1	2	31-Aug-18
<=8 hours	1	1	<=8 hours	2.5	1	2	1-Sep-18
<=8 hours	1	1	<=8 hours	1	0	0	4-Sep-18
<=8 hours	0	1	<=8 hours	0	0	0	4-Sep-18
<=8 hours	1	1	<=8 hours	6	1	3	4-Sep-18
<=8 hours	1	1	<=8 hours	7	1	0	5-Sep-18
		0					6-Sep-18
<=8 hours	1	1	<=8 hours	0		0	6-Sep-18
<=8 hours	1	1	<=8 hours	0	0	0	6-Sep-18
<=8 hours	1	1	<=8 hours	2	1	4	6-Sep-18
<=8 hours	1	1	<=8 hours	1	1	3	6-Sep-18
<=8 hours	1	1	>8 hours	2	1	9	6-Sep-18
>8 hours	1	1	<=8 hours	9.5	1	1	6-Sep-18
<=8 hours	1	1	<=8 hours	4.5	1	0	6-Sep-18
<=8 hours	1	1	<=8 hours	0	0	0	5-Sep-18
>8 hours	1	1	>8 hours	11	1	11	5-Sep-18
>8 hours	1	1	>8 hours	9.5	1	10	5-Sep-18
<=8 hours	1	1	>8 hours	5	1	9	6-Sep-18
>8 hours	1	1	>8 hours	10	1	8.5	24-Sep-18
>8 hours	1	1	>8 hours	13	1	12	24-Sep-18
<=8 hours	1	1	<=8 hours	5	1	5.5	25-Sep-18
>8 hours	1	1	<=8 hours	10.5	1	5	25-Sep-18
>8 hours	1	1	<=8 hours	9	0	0	22-Sep-18
>8 hours	1	1	<=8 hours	13	0	0	22-Sep-18
<=8 hours	0	1	<=8 hours	0	0	0	15-Sep-18
<=8 hours	1	1	<=8 hours	0	1	0	26-Sep-18
<=8 hours	0	1	<=8 hours	0	0	0	26-Sep-18

<=8 hours	1	1	<=8 hours	4	1	2.5	26-Sep-18
<=8 hours	1	1	>8 hours	3	1	9	27-Sep-18
>8 hours	1	1	<=8 hours	9.5	1	6.5	27-Sep-18
>8 hours	1	1	>8 hours	9	1	8	27-Sep-18
>8 hours	1	1	<=8 hours	10	1	7	27-Sep-18
<=8 hours	1	1	>8 hours	5	1	9	27-Sep-18
<=8 hours	1	1	<=8 hours	0	0	0	27-Sep-18
>8 hours	1	1	>8 hours	14	1	8	27-Sep-18
>8 hours	1	1	<=8 hours	9	1	3	27-Sep-18
<=8 hours	1	0	<=8 hours	0	1	0	25-Sep-18
>8 hours	1	1	<=8 hours	11	1	0	28-Sep-18
<=8 hours	1	1	<=8 hours	1.8	1	4	28-Sep-18
<=8 hours	1	1	<=8 hours	4	1	0	27-Sep-18
<=8 hours	1	1	<=8 hours	2	1	1	14-Oct-18
<=8 hours	1	1	>8 hours	7	1	8	15-Oct-18
>8 hours	1	1	>8 hours	14	1	9	15-Oct-18
>8 hours	1	1	<=8 hours	11	0	0	18-Oct-18
<=8 hours	1	1	<=8 hours	0	1	0	20-Oct-18
<=8 hours	0	1	<=8 hours	0	0	0	24-Oct-18
<=8 hours	1	1	<=8 hours	5.5	1	2.5	26-Oct-18
<=8 hours	1	1	<=8 hours	4	1	0	26-Oct-18
<=8 hours	1	0	<=8 hours	0	1	0	26-Oct-18
>8 hours	1	1	>8 hours	11.5	1	14.5	26-Oct-18
<=8 hours	1	1	>8 hours	4	1	8.5	10-Oct-18
<=8 hours	1	1	<=8 hours	0	0	0	10-Oct-18
<=8 hours	1	1	>8 hours	4.5	1	13.5	20-Oct-18
>8 hours	1	1	>8 hours	14.5	1	12.5	22-Oct-18
>8 hours	1	1	>8 hours	12	1	10	22-Oct-18
<=8 hours	1	1	<=8 hours	6	1	6	27-Oct-18
<=8 hours	1	0	<=8 hours	0	1	0	26-Oct-18
>8 hours	1	1	<=8 hours	10	1	6	30-Oct-18
>8 hours	1	1	>8 hours	8	1	8	30-Oct-18
<=8 hours	1	1	<=8 hours	7	1	2	2-Nov-18
<=8 hours	1	1	<=8 hours	6	1	2	30-Oct-18
<=8 hours	1	1	>8 hours	4	1	9	30-Oct-18
<=8 hours	1	1	<=8 hours	0	1	0	2-Nov-18
<=8 hours	1	1	<=8 hours	3	1	5.5	2-Nov-18
>8 hours	1	1	<=8 hours	10	1	6.5	4-Nov-18
>8 hours	1	1	<=8 hours	10	1	4	4-Nov-18
	1	1					9-Nov-18

ldateifcontinuing	DatoofDaysKM	llowuptoho	terviewEBF	usDayofInt	ayscoloured	hosp_cat	17.M_Agey	MEducation
		0	1	1	3	<3 day	23	9
		0	1	1	3	<3 day	23	9
				1	3	<3 day	23	12
			1	1	3	<3 day	20	9
		0	1	1	15	>3 days	28	8
		0	1	1	3	<3 day	28	0
			1	1	7	>3 days	27	17
		1	1	1	3	<3 day	30	12
			1	1	7	>3 days	20	0
			1	1	7	>3 days	20	0
			1	1	15	>3 days	22	2
			1	1	3	<3 day	20	8
		1	1	1	2	<3 day	20	3
		0	1	1	3	<3 day	19	5
		0	1	1	1	<3 day	27	2
		1	1	1	3	<3 day	25	0
		1	1	1	6	>3 days	21	4
		1	0	1	15	>3 days	27	12
		0	1	1	4	>3 days	35	5
		1	1	1	3	<3 day	25	0
17-Feb-18	24	0	1	1	11	>3 days	20	12
17-Feb-18	30	0	1	1	3	<3 day	20	0
19-Feb-18	35	1	1	1	3	<3 day	24	0
20-Feb-18	34	1	1	1	3	<3 day	26	5
20-Feb-18	32	1	1	1	3	<3 day	19	8
26-Jan-18	8	1	1	0	3	<3 day	20	0
18-Feb-18	34	1	0	1	3	<3 day	24	0
			1	1	6	>3 days	17	8
		1	1	1	10	>3 days	21	11
		1	1	1	8	>3 days	22	15
		1	1	1	3	<3 day	25	0
		0	1	1	3	<3 day	20	4
		1	1	1	10	>3 days	19	0
		1	1	1	6	>3 days	19	0
			1	1	4	>3 days	25	10
		1	1	1	2	<3 day	25	15
		1	1	1	3	<3 day	33	0
		1	1	1	15	>3 days	30	2
			0	1	6	>3 days	19	9
		1	1	1	6	>3 days	24	1
14-Mar-18	32	0	1	1	3	<3 day	24	0
21-Mar-18	30		1	1	3	<3 day	26	12
14-Mar-18	35	1	0	1	12	>3 days	30	7
16-Mar-18	39	0	1	1	9	>3 days	22	0
14-Mar-18	31	1	0	1	8	>3 days	27	14
		0	1	1	3	<3 day	23	12

		1	0	1	3	<3 day	24	10
		1	1	1	8	>3 days	24	10
		1	1	1	5	>3 days	22	1
11-Apr-18	45	1	1	1	10	>3 days	23	15
11-Apr-18	33	1	1	1	9	>3 days	21	14
		1	1	1	10	>3 days	21	14
		1	1	1	6	>3 days	21	9
		1	1	1	3	<3 day	28	0
		0	1	1	2	<3 day	22	7
		0	1	1	10	>3 days	21	10
		1	1	1	7	>3 days	26	0
		0	1	1	2	<3 day	21	8
		0	1	1	3	<3 day	20	7
		0	1	1	3	<3 day	22	10
		0	1	1	0	<3 day	18	0
		1	1	1	11	>3 days	30	7
30-Mar-18	19	0	1	1	3	<3 day	19	7
		1	1	1	13	>3 days	19	10
		1	1	1	2	<3 day	21	7
		0	1	1	3	<3 day	21	10
		1	1	1	14	>3 days	28	15
		1	1	1	7	>3 days	23	7
		1	1	1	7	>3 days	20	0
		0	1	1	5	>3 days	20	4
		1	1	1	3	<3 day	18	7
		0	1	1	2	<3 day	21	15
		1	1	1	3	<3 day	18	0
		1	1	1	1	<3 day	20	8
		0	1	1	3	<3 day	25	0
			1	1	10	>3 days	26	17
			1		18	>3 days	26	17
20-Apr-18	24	0	1	1	3	<3 day	23	2
29-May-18	40	1	1	1	4	>3 days	24	10
13-May-18	21	0	1	1	3	<3 day	20	5
7-Jun-18	22	0	1	1	2	<3 day	24	10
8-Jun-18	25	0	1	1	7	>3 days	25	4
7-Jun-18	18		1	1	8	>3 days	19	8
8-Jun-18	44	0	1	1	1	<3 day	23	4
5-Jun-18	31	1	1	1	5	>3 days	20	10
7-Jun-18	43	1	1	1	6	>3 days	20	0
7-Jun-18	41	1	1	1	3	<3 day	26	0
7-Jun-18		1	0		30	>3 days	26	5
7-Jun-18	29	1	1	1	5	>3 days	24	10
7-Jun-18	34	0	1	1	2	<3 day	25	0
7-Jun-18	42	1	1	1	11	>3 days	27	17
8-Jun-18	39	1	1	1	12	>3 days	24	10
1-Jun-18	37	1	1	1	8	>3 days	25	17

28-May-18	25	1	0	1	3	<3 day	28	12
20-May-18	31	1	1	1	9	>3 days	21	10
8-Jun-18	34	1	0		15	>3 days	20	9
8-Jun-18	34	1	0	1	15	>3 days	20	9
20-May-18	28	0	1	1	3	<3 day	22	0
28-May-18	18	0	1	1	3	<3 day	33	12
2-Jun-18	24	0	1	1	3	<3 day	20	4
5-Jul-18	25	1	1		4	>3 days	22	10
5-Jul-18	28	1	0	1	3	<3 day	50	13
21-Jun-18	20	1	1	1	3	<3 day	19	10
5-Jul-18	35	1	1	1	12	>3 days	20	10
5-Jul-18	35	1	1	1	12	>3 days	20	10
5-Jul-18	31	1	0	1	3	<3 day	20	10
5-Jul-18	31	1	0	1	6	>3 days	20	10
5-Jul-18	29	1	1	1	6	>3 days	19	7
5-Jul-18	27	1	1	1	3	<3 day	21	7
29-Jun-18	46	0	1	1	6	>3 days	22	3
20-Jun-18	30	1	1	1	3	<3 day	21	10
6-Jul-18	24	1	1	1	8	>3 days	25	0
6-Jul-18	35	1	1	1	4	>3 days	18	8
6-Jul-18	15	1	1	1	5	>3 days	20	0
6-Jul-18	24	1	1	1	5	>3 days	25	10
6-Jul-18	24	0	1	1	3	<3 day	20	7
6-Jul-18	34	1	1	1	8	>3 days	21	10
23-Apr-18	21	0	1	1	3	<3 day	18	0
5-Jul-18	35	1	1	1	5	>3 days	31	15
11-Jul-18	18	1	1	1	10	>3 days	22	8
11-Jul-18	18	1	1	1	10	>3 days	22	8
16-Jul-18	46	0	1	1	3	<3 day	28	0
16-Jul-18	46	0	1	1	3	<3 day	28	0
8-Jul-18	23	0	1	1	3	<3 day	24	14
18-Jul-18	28	0	1	1	3	<3 day	30	7
		0	1	1	6	>3 days	20	10
25-Jul-18	21	1	1	1	10	>3 days	20	7
26-Jul-18	34	1	1	1	8	>3 days	28	7
26-Jul-18	34	1	1	1	8	>3 days	28	7
3-Aug-18	16	1	1	1	3	<3 day	25	0
7-Jul-18	46	1	1	1	5	>3 days	24	7
3-Aug-18	34	1	1	1	8	>3 days	23	8
8-Aug-18	28	1	1	1	3	<3 day	21	7
8-Aug-18	36	1	1	1	3	<3 day	18	2
6-Aug-18	29	0	1	1	3	<3 day	19	8
9-Aug-18	27	1	1	1	15	>3 days	20	7
9-Aug-18	40	1	0	1	4	>3 days	20	5
25-Jul-18	22	0	1	1	3	<3 day	19	7
9-Aug-18	40	0	1	1	3	<3 day	24	5
3-Aug-18	51	1	1	1	2	<3 day	23	4

9-Aug-18	40	0	1	1	3	<3 day	22	6
9-Aug-18	32	1	1	1	10	>3 days	22	8
9-Aug-18	42	1	1	1	2	<3 day	26	8
7-Aug-18	27	1	1	1	4	>3 days	28	2
7-Aug-18	43	1	1	1	10	>3 days	24	3
8-Aug-18	30	1	1	1	6	>3 days	20	8
8-Aug-18	21	1	1	1	3	<3 day	27	0
10-Aug-18	13	1	1	1	12	>3 days	26	12
10-Aug-18	20	1	1	1	9	>3 days	22	7
21-Jul-18	26	0	1	1	3	<3 day	22	9
10-Aug-18	23	0	1	1	2	<3 day	22	10
18-Aug-18	24	0	1	1	26	>3 days	20	3
19-Aug-18	25	0	1	1	19	>3 days	23	8
19-Aug-18	45	1	1	1	20	>3 days	20	0
22-Aug-18	26	1	1	1	8	>3 days	20	5
22-Aug-18	27	1	1	1	8	>3 days	20	5
7-Aug-18	2	0	1	1	4	>3 days	29	7
22-Aug-18	31	0	1	1	3	<3 day	21	12
31-Aug-18	28	0	1	1	11	>3 days	27	12
31-Aug-18	28	0	1	1	11	>3 days	27	12
31-Aug-18	40	0	0	1	3	<3 day	22	8
30-Aug-18	35	1	1	1	2	<3 day	23	10
11-Sep-18	40	0	1	1	1	<3 day	26	4
11-Sep-18	40	0	1	1	1	<3 day	26	4
4-Sep-18	36	0	1	1	3	<3 day	30	4
5-Sep-18	33	1	1	1	3	<3 day	22	9
31-Aug-18	28	1	1	1	7	>3 days	31	17
6-Sep-18	40	1	1	1	6	>3 days	21	2
6-Sep-18	31	1	1	1	14	>3 days	31	14
6-Sep-18	35	1	1	1	8	>3 days	26	7
6-Sep-18	35	1	1	1	8	>3 days	26	7
6-Sep-18	29	0	1	1	3	<3 day	20	4
3-Sep-18	36	1	1	1	3	<3 day	20	10
6-Sep-18	29	0	1	1	3	<3 day	30	2
3-Sep-18	31	0	1	1	5	>3 days	21	7
5-Sep-18	22	1	1	1	14	>3 days	25	0
5-Sep-18	36	1	1	1	19	>3 days	27	10
6-Sep-18	25	0	1	1	8	>3 days	30	15
24-Sep-18	32	1	1	1	8	>3 days	23	11
24-Sep-18	37	1	1	1	18	>3 days	22	5
25-Sep-18	33	0	1	1	3	<3 day	26	7
23-Sep-18	27	1	1	1	7	>3 days	28	8
22-Sep-18	22	0	1	1	10	>3 days	25	7
22-Sep-18	22	0	1	0	10	>3 days	25	7
1-Sep-18	17	0	1	1	5	>3 days	20	10
26-Sep-18	18	1	1	1	8	>3 days	25	2
26-Sep-18	18	0	1	1	9	>3 days	29	7

26-Sep-18	25	1	1	1	7	>3 days	24	10
27-Sep-18	17	1	1	1	9	>3 days	18	5
27-Sep-18	29	0	1	1	5	>3 days	22	10
27-Sep-18	20	1	1	1	6	>3 days	20	5
27-Sep-18	30	0	1	1	3	<3 day	22	10
27-Sep-18	42	0	1	1	3	<3 day	20	5
27-Sep-18	37	1	1	1	8	>3 days	26	8
27-Sep-18	32	1	1	1	9	>3 days	25	0
27-Sep-18	23	0	1	1	7	>3 days	20	5
25-Sep-18	33	1	1	1	6	>3 days	22	15
28-Sep-18	29	0	1	1	3	<3 day	23	5
28-Sep-18	39	1	1	1	6	>3 days	25	10
21-Sep-18	21	1	1	1	2	<3 day	22	3
30-Sep-18	18	1	1	1	3	<3 day	24	12
15-Oct-18	31	1	0	1	3	<3 day	27	6
15-Oct-18	31	1	0	1	3	<3 day	27	6
18-Oct-18	31	0	1	1	4	>3 days	25	3
20-Oct-18	39	0	1	1	9	>3 days	28	0
24-Oct-18	31	1	1	1	20	>3 days	28	10
16-Oct-18	26	0	0	1	3	<3 day	25	0
26-Oct-18	29	1	1	1	7	>3 days	24	5
26-Oct-18	22	1	1	1	5	>3 days	22	6
24-Oct-18	30	1	1	1	8	>3 days	20	8
10-Oct-18	27	0	1	1	2	<3 day	30	10
10-Oct-18	24	0	1	1	3	<3 day	27	12
20-Oct-18	27	0	1	1	8	>3 days	20	0
22-Oct-18	23	0	1	1	28	>3 days	21	10
18-Oct-18	38	0	1	1	4	>3 days	22	5
15-Oct-18	5	0	1	1	5	>3 days	27	7
15-Oct-18	21	0	1	1	3	<3 day	24	7
30-Oct-18	48	0	1	1	3	<3 day	24	0
25-Oct-18	32	0	1	1	3	<3 day	20	2
28-Oct-18	38	0	1	1	3	<3 day	25	0
30-Oct-18	41	1	1	1	2	<3 day	28	7
30-Oct-18	41	1	1	1	2	<3 day	28	7
2-Nov-18	38	1	1	1	4	>3 days	22	12
2-Nov-18	40	1	1	1	6	>3 days	21	3
27-Oct-18	34	1	1	1	3	<3 day	30	10
27-Oct-18	34	1	1	1	3	<3 day	30	10
5-Nov-18	35	1	1	1	11	>3 days	23	0

Meducat	skilled2Busi	ation1.Skilled2unskilled3	M_AgeofSp	_Education	.i.M_OccSp	_SpouseO	.M_NoofCh
>8	2	Farmer		9.00	Labour	2	1
>8	2	Farmer		9.00	Labour	2	1
>8	1	Tailor		10.00	Driver	1	0
>8	2	Farmer		3.00	Farmer	2	0
>8	1	Bangle making		10.00	Driver	1	1
<8	2	Farming		0.00	Farming	2	2
>8	4	At home		17.00	ank manage	1	0
>8	1	School teacher		9.00	Factory	1	1
<8	2	Farmer		0.00	Labourer	2	0
<8	2	Farmer		0.00	Labourer	2	0
<8	2	Farming		0.00	Farming	2	0
>8	2	Farming		0.00	Farming	2	1
<8	2	Farming		8.00	Driver lorry	1	0
<8	2	farming		10.00	Supervisor	1	1
<8	2	Farming		0.00	Farming	2	3
<8	4	At home		8.00	Auto driver	1	2
<8	2	Mill		10.00	ertiliser sho	2	1
>8	4	At home		15.00	Dairy	1	0
<8	2	Farming		0.00	Farming	2	0
<8	1	Garment factory		12.00	MC Machin	1	0
>8	4	house wife		13.00	Farmer	2	0
<8	2	Coolie		0.00	Coolie	2	1
<8	2	Coolie		12.00	Coolie	2	1
<8	4	house wife		1.00	Coolie	2	2
>8	2	Coolie		7.00	Coolie	2	0
<8	2	Coolie		6.00	Coolie	2	0
<8	2	Coolie		0.00	Coolie	2	2
>8	2	Coolie		0.00	Coolie	2	0
>8	4	At home		7.00	Driver	1	0
>8	4	At home		17.00	eacher in go	1	0
<8	4	At home		0.00	Coolie	2	2
<8	2	Farming		0.00	Farming	2	0
<8	2	Farming		9.00	Farming	2	1
<8	2	Coolie		0.00	Coolie	2	0
>8	4	At home		12.00	Farming	2	0
>8	4	At home		15.00	Farming	2	1
<8	2	Sells clothes, vessels		1.00	clothes, ve	2	0
<8	2	Coolie		0.00	Shepherd	2	3
>8	4	At home		0.00	)river tractc	1	0
<8	2	farming		0.00	Farming	2	1
<8	2	Coolie		7.00	Farming	2	1
>8	4	house wife	32	15.00	Farming	2	2
<8	4	house wife		15.00	Carpenter	1	1
<8	2	Coolie	25	5.00	Coolie	2	2
>8	1	teacher		14.00	Engineer	1	1
>8	4	At home	27	7.00	Coolie	2	0

>8	2	Flowermaking	30	15.00	Florist	2	0
>8	2	Flowermaking	30	15.00	Florist	2	0
<8	2	Coolie	27	0.00	Coolie	2	2
>8	4	At home	30	19.00	eacher in go	1	0
>8	4	At home	28	14.00	lkworm Far	2	0
>8	4	At home	26	12.00	nplyed Pvt (	1	0
>8	4	At home	25	15.00	Supplier	1	0
<8	2	Coolie	30	0.00	Farming	2	1
<8	2	Coolie	26	0.00	river tractc	1	2
>8	4	At home	27	10.00	car driver	1	0
<8	2	Coolie	28	0.00	Coolie	2	3
>8	4	At home	23	10.00	Cook	2	0
<8	4	At home	25	0.00	farming	2	1
>8	4	At home	24	8.00	Supervisor	1	1
<8	2	stitches	30	0.00	farming	2	0
<8	4	At home	33	15.00	Farming	2	0
<8	4	At home	26	10.00	Labour	2	0
>8	4	At home	24	9.00	Driver Lorry	1	0
<8	4	At home	24	0.00	Coolie	2	0
>8	4	At home	26	10.00	Coolie	2	0
>8	1	accountant	29	13.00	Contractor	2	0
<8	2	Coolie	25	0.00	Coolie	2	1
<8	2	Coolie	24	0.00	Driver	1	0
<8	2	Coolie	28	0.00	Coolie	2	1
<8	2	Farmer	22	8.00	Auto driver	1	0
>8	4	At home	27	15.00	ectrical PVt	1	0
<8	2	Farmer	24	8.00	Farmer	2	0
>8	2	Farmer	22	9.00	Farmer	2	0
<8	2	Coolie	27	6.00	Coolie	2	0
>8	1	teacher	30	18.00	Bank Mana	1	0
>8	1	teacher	30	18.00	Bank Mana	1	0
<8	2	Labourer	30	4.00	Labourer	2	0
>8	4	house wife	30	12.00	Farmer	2	1
<8	2	Labourer	25	5.00	Labourer	2	0
>8	2	Coolie	26	0.00	Coolie	2	0
<8	2	Labourer	30	0.00	Labourer	2	1
>8	4	At home	25	12.00	eacher in go	1	0
<8	2	Labourer	25	12.00	Labourer	2	1
>8	2	Optical Sales girl	26	0.00	Labourer	2	0
<8	2	Labourer	30	0.00	Labourer	2	0
<8	2	Coolie	33	0.00	Coolie	2	1
<8	2	Labourer	28	0.00	Labourer	2	2
>8	4	At home	28	12.00	Farming	2	1
<8	2	Devadasi					1
>8	4	At home	29	15.00	usiness Stor	3	0
>8	2	Coolie	26	6.00	Coolie	2	1
>8	1	teacher	27	15.00	Paper agent	3	1

>8	4	At home	35	0.00	Labourer	2	1
>8	1	Receptionist	28	13.00	welers Labc	2	1
>8	4	At home	23	8.00	Mason	2	0
>8	4	At home	23	8.00	Mason	2	0
<8	2	Labourer	38	0.00	Labourer	2	0
>8	1	Anganwadi teacher	36	12.00	Farmer	2	1
<8	2	Labourer	25	5.00	Labourer	2	0
>8	4	At home	31	10.00	Coolie	2	1
>8	4	At home	62	10.00	Labourer	2	0
>8	4	At home	27	10.00	Mechanic	1	0
>8	4	At home	25	11.00	Farming	2	0
>8	4	At home	25	11.00	Farming	2	0
>8	4	At home	25	10.00	Mason	2	0
>8	4	At home	25	10.00	Mason	2	0
<8	2	Coolie	20	7.00	Coolie	2	0
<8	2	Farming	25	12.00	Farming	2	0
<8	2	Farming	25	0.00	Farming	2	0
>8	4	At home	30	14.00	ectrical PVt	1	0
<8	2	Labourer		0.00	Labourer	2	1
>8	2	Farming	25	10.00	Farming	2	0
<8	2	Farming	23	10.00	Farming	2	0
>8	4	At home	31	12.00	Bank	1	0
<8	2	Farming	22	0.00	Farming	2	0
>8	4	At home	31	7.00	Bakery shop	3	0
<8	2	Coolie	24	0.00	Coolie	2	0
>8	1	teacher	30	17.00	ank manage	1	0
>8	2	Farming	28	0.00	Labour	2	0
>8	2	Farming	28	0.00	Labour	2	0
<8	4	house wife	30	0.00	Labour	2	2
<8	4	house wife	30	0.00	Labour	2	2
>8	1	teacher	30	17.00	Business	3	0
<8	4	house wife	35	0.00	Business	3	2
>8	2	Farmer	22	0.00	Farmer	2	0
<8	4	house wife	23	7.00	Driver	1	0
<8	2	farming	32	0.00	Farming	2	2
<8	2	farming	32	0.00	Farming	2	2
<8	4	house wife	27	0.00	Labour	2	0
<8	4	house wife	28	10.00			0
>8	4	house wife	26	4.00	our supervi	2	0
<8	4	house wife	28	6.00	labourer	2	0
<8	4	house wife	30	0.00	driver	1	0
>8	4	house wife	23	10.00	conductor	2	1
<8	1	tailoring	28	2.00	struction wc	2	0
<8	2	farming	22	6.00	farming	2	0
<8	2	Farming	25	7.00	farming	2	2
<8	2	labourer	26	0.00	Labourer	2	1
<8	4	house wife	23	7.00	uide for Han	2	0

<8	2	Coolie	25	0.00	Driver	1	2
>8	4	house wife	30	7.00	Farmer	2	1
>8	2	Coolie	30	2.00	Coolie	2	2
<8	2	Farming	32	12.00	Farming	2	1
<8	2	Coolie	30	5.00	Coolie	2	0
>8	2	Coolie	23	8.00	Coolie	2	0
<8	2	Coolie	45	2.00	Coolie	2	1
>8	4	house wife	30	10.00	Farmer	2	2
<8	2	Farming	27	10.00	Farming	2	0
>8	4	house wife	25	0.00	Farmer	2	0
>8	2	Coolie	28	12.00	Coolie	2	1
<8	2	Farming	24	6.00	Farming	2	0
>8	2	Labourer	30	0.00	Labour	2	1
<8	2	Labourer	25	4.00	Labour	2	0
<8	2	Labourer	23	12.00	Labour	2	0
<8	2	Labourer	23	12.00	Labour	2	0
<8	4	house wife	32	12.00	Auto driver	1	2
>8	4	house wife	27	14.00	mastri	2	0
>8	4	house wife	36	12.00	Business	3	2
>8	4	house wife	36	12.00	Business	3	2
>8	4	house wife	25	7.00	Driver	1	0
>8	2	Coolie	28	9.00	Farmer	2	0
<8	4	house wife	30	6.00	Farmer	2	2
<8	4	house wife	30	6.00	Farmer	2	2
<8	4	house wife	35	10.00			3
>8	2	Coolie	25	3.00	Coolie	2	0
>8	1	teacher	32	15.00	entry Pancl	1	1
<8	2	Coolie	24	10.00	Farming	2	0
>8	1	teacher	32	12.00	Business	3	1
<8	4	At home	35	0.00	cylinder de	2	0
<8	4	At home	35	0.00	cylinder de	2	0
<8	2	Coolie	30	0.00	Coolie	2	0
>8	2	Farming	23	10.00	Farming	2	0
<8	2	Labourer	32	0.00	ractor drive	1	1
<8	2	farming	26	10.00	Milk vendo	3	1
<8	2	farming	30	0.00	Farming	2	1
>8	2	Snack shop	35	10.00	Snack shop	3	1
>8	4	At home	33	10.00	lway emplo	1	1
>8	4	At home	33	12.00	Farmer	2	0
<8	4	At home	28	15.00	Teacher	1	0
<8	2	Labourer	38	0.00	Farmer	2	2
>8	4	house wife	32	5.00	Farmer	2	3
<8	2	Labourer	30	0.00	dairy labo	2	1
<8	2	Labourer	30	0.00	dairy labo	2	1
>8	4	At home	25	12.00	Business	3	0
<8	4	At home	35	15.00	Teacher	1	1
<8	4	At home	33	7.00	struction wo	2	2

>8	4	At home	28	12.00	achine cutt	1	1
<8	2	Coolie	25	5.00	Shop	3	0
>8	4	At home	26	6.00	Farming	2	1
<8	2	Farming	24	0.00	Farming	2	0
>8	4	At home	28	0.00	Farming	2	1
<8	2	Labourer	28	6.00	Farming	2	2
>8	2	Farming	30	7.00	ractor drive	1	1
<8	2	Farming	30	0.00	Farming	2	0
<8	2	Farming	23	0.00	Farming	2	0
>8	4	At home	26	12.00	Farming	2	0
<8	2	Coolie	24	10.00	Farming	2	0
>8	4	At home	30	15.00	uter operat	1	0
<8	2	farming	24	12.00	electrician	1	2
>8	4	At home	28	12.00	Farming	2	1
<8	2	Coolie	32	0.00	Driver	1	2
<8	2	Coolie	32	0.00	Driver	1	2
<8	2	Labourer	30	0.00	Labourer	2	1
<8	2	Coolie	32	2.00	Farming	2	1
>8	1	ASHA worker	35	7.00	Mesthri	2	2
<8	2	Coolie	30	0.00	Coolie	2	1
<8	2	Coolie	28	9.00	Farming	2	0
<8	2	Farming	25	0.00	Farming	2	0
>8	4	At home	25	10.00	Mechanic	1	0
>8	1	Beautician	35		Business	3	1
>8	4	At home	30	15.00	Farming	2	0
<8	4	At home	25	0.00	Farming	2	0
>8	2	labourer	27	0.00	Labourer	2	0
<8	4	At home	25	12.00	Farmer	2	0
<8	4	At home	35	0.00	Farming	2	1
<8	4	At home	30	4.00	Farming	2	1
<8	2	labourer	26	4.00	Farming	2	3
<8	2	labourer	25	0.00	Labourer	2	0
<8	2	labourer	30	8.00	Labourer	2	1
<8	2	Farmer	30	10.00	labourer	2	0
<8	2	Farmer	30	10.00	labourer	2	0
>8	2	Farmer	27	5.00	Labourer	2	1
<8	1	tailoring	23	10.00	Farming	2	0
>8	2	labourer	32	7.00	Farming	2	3
>8	2	labourer	32	7.00	Farming	2	3
<8	4	At home	26	0.00	Business	3	5

ildrenPrim	owledge_TC	tQ127Y10	TQ137BA1	SupIniKMC	tQ14BI1or	15.KMCKit	18.Y5No0	tenanceHo
2	8	3	3	6	3	5	5	13
2	8	3	3	6	3	5	5	13
1	13	3	3	6	3	0	5	8
1	20	5	3	8	3	5	0	8
2	14	3	3	6	3	0	0	3
2	16	3	3	6	3	0	0	3
1	16	5	3	8	3	5	0	8
2	16	3	3	6	3	0	0	3
1	16	3	3	6	3	5	5	13
1	16	3	3	6	3	5	5	13
1	16	3	3	6	3	5	0	8
2	18	3	3	6	3	5	0	8
1	13	0	3	3	0	0	0	0
2	17	3	3	6	3	0	0	3
2	15	3	3	6	3	0	0	3
2	11	3	3	6	3	0	0	3
2	14	0	0	0	0	0	0	0
1	13	3	3	6	3	5	0	8
1	13	0	0	0	3	0	0	3
1	16	0	3	3	3	0	0	3
1	21	3	3	6	3	0	5	8
2	14	3	3	6	3	0	0	3
2	17	3	3	6	3	0	0	3
2	15	3	3	6	3	0	0	3
1	19	3	3	6	3	5	0	8
1	21	3	3	6	3	5	0	8
2	16	3	3	6	3	5	0	8
1	16	3	3	6	3	0	0	3
1	23	5	5	10	0	5	0	5
1	15	3	3	6	3	5	0	8
2	16	0	3	3	0	0	0	0
1	15	3	3	6	3	5	0	8
2	15	3	3	6	3	5	0	8
1	15	3	3	6	3	5	0	8
1	12	3	3	6	3	5	0	8
2	15	0	0	0	0	0	0	0
1	16	3	3	6	3	5	5	13
2	13	3	3	6	3	0	0	3
1	12	3	3	6	3	5	5	13
2	16	0	3	3	0	0	0	0
2	21	3	3	6	3	0	0	3
2	20	3	3	6	3	5	0	8
2	18	3	3	6	3	5	0	8
2	20	3	3	6	3	5	5	13
2	22	3	3	6	3	5	0	8
1	18	3	3	6	3	0	0	3

1	20	3	3	6	4	5	5	14
1	20	3	3	6	4	5	5	14
2	14	3	3	6	3	0	0	3
1	17	3	3	6	3	5	0	8
1	19	3	3	6	3	0	0	3
1	16	3	3	6	3	5	0	8
1	16	3	3	6	3	5	0	8
2	14	3	3	6	3	0	0	3
2	16	3	3	6	3	0	0	3
1	16	3	3	6	3	5	0	8
2	19	3	3	6	3	5	0	8
1	15	3	3	6	3	0	0	3
2	16	3	3	6	3	5	0	8
2	17	3	5	8	3	5	5	13
1	13	3	3	6	3	5	0	8
1	19	3	3	6	3	5	0	8
1	19	3	3	6	3	5	0	8
1	16	3	3	6	3	5	0	8
1	19	3	3	6	3	5	5	13
1	15	3	3	6	3	0	0	3
1	19	3	3	6	3	0	0	3
2	17	3	3	6	3	5	0	8
1	15	3	3	6	4	0	0	4
2	14	3	5	8	3	0	0	3
1	17	3	5	8	3	5	0	8
1	17	3	3	6	3	0	0	3
1	12	3	3	6	3	5	5	13
1	18	3	3	6	0	0	0	0
1	14	3	3	6	3	5	5	13
1	22	3	3	6	3	5	5	13
1	22	3	3	6	3	5	5	13
1	11	3	3	6	3	5	0	8
2	20	3	3	6	3	5	0	8
1	21	3	5	8	3	5	0	8
1	18	0	3	3	0	5	0	5
2	13	5	5	10	3	5	0	8
1	20	3	3	6	0	0	0	0
2	12	5	5	10	3	5	5	13
1	22	3	5	8	3	5	0	8
1	16	3	3	6	3	5	0	8
2	16	3	3	6	3	5	0	8
2	15	3	3	6	3	5	5	13
2	15	0	3	3	3	0	0	3
2	13	3	3	6	3	0	0	3
1	17	5	5	10	3	5	5	13
2	17	5	5	10	3	5	0	8
2	18	3	3	6	3	5	0	8

2	15	5	5	10	3	0	0	3
2	19	3	3	6	3	5	0	8
1	15	5	5	10	3	5	5	13
1	15	5	5	10	3	5	5	13
1	14	5	3	8	4	0	0	4
2	22	0	3	3	0	0	0	0
1	14	0	3	3	0	0	0	0
2	18	3	3	6	3	5	0	8
1	17	3	3	6	3	5	0	8
1	15	3	3	6	3	5	0	8
1	19	5	3	8	3	5	5	13
1	19	5	3	8	3	5	5	13
1	18	5	5	10	3	5	0	8
1	18	5	5	10	3	5	0	8
1	16	3	5	8	3	5	5	13
1	15	5	5	10	3	5	5	13
1	17	5	5	10	3	5	0	8
1	19	3	3	6	3	0	0	3
2	14	0	0	0	0	5	0	5
1	12	3	3	6	3	5	5	13
1	11	0	0	0	0	0	0	0
1	17	3	5	8	3	0	5	8
1	15	5	5	10	3	5	5	13
1	15	5	3	8	3	5	0	8
1	19	3	3	6	4	5	0	9
1	19	0	5	5	0	5	5	10
1	21	3	5	8	5	5	0	10
1	21	3	5	8	5	5	0	10
2	16	3	5	8	4	5	0	9
2	16	3	5	8	4	5	0	9
1	20	0	0	0	0	0	0	0
2	16	3	3	6	4	5	0	9
1	21	3	3	6	3	5	0	8
1	24	3	3	6	4	5	5	14
2	22	3	3	6	4	5	0	9
2	22	3	3	6	4	5	0	9
1	19	3	5	8	4	5	5	14
1	16	3	3	6	3	0	0	3
1	21	3	3	6	3	5	5	13
1	17	3	5	8	4	5	5	14
1	17	5	3	8	3	5	5	13
2	24	3	3	6	3	5	0	8
1	22	5	5	10	3	5	5	13
1	12	0	0	0	0	5	5	10
2	21	5	5	10	3	5	0	8
2	16	5	5	10	4	5	0	9
1	18	3	3	6	3	5	0	8

2	18	5	5	10	3	0	0	3
2	20	3	3	6	3	5	0	8
2	23	3	3	6	3	5	0	8
2	21	5	3	8	4	5	0	9
1	13	5	3	8	0	5	0	5
1	21	5	5	10	3	5	0	8
2	16	3	5	8	4	5	0	9
2	18	3	5	8	5	5	5	15
1	16	3	5	8	3	0	0	3
1	20	5	3	8	3	5	0	8
2	22	3	5	8	5	5	0	10
1	21	3	3	6	3	5	0	8
2	23	3	3	6	3	5	0	8
1	19	5	5	10	3	5	5	13
1	21	5	3	8	4	5	0	9
1	21	5	3	8	4	5	0	9
2	10	3	3	6	3	5	0	8
1	21	3	3	6	3	5	0	8
2	23	3	3	6	4	5	0	9
2	23	3	3	6	4	5	0	9
1	19	3	3	6	4	5	0	9
1	19	3	3	6	3	5	0	8
2	18	3	3	6	3	5	5	13
2	18	3	3	6	3	5	5	13
2	21	3	3	6	3	5	0	8
1	18	3	3	6	3	5	0	8
2	17	0	0	0	0	5	5	10
1	16	3	5	8	3	5	5	13
2	21	3	3	6	3	5	5	13
1	18	3	3	6	3	5	5	13
1	18	3	3	6	3	5	5	13
1	14	3	5	8	4	5	0	9
1	19	5	3	8	4	5	0	9
2	15	3	5	8	3	5	0	8
2	13	3	3	6	3	5	0	8
2	16	3	3	6	3	5	0	8
2	16	3	3	6	3	5	0	8
2	19	5	5	10	3	5	0	8
1	17	3	3	6	3	5	0	8
1	23	3	3	6	4	5	5	14
2	21	3	3	6	3	5	0	8
2	18	5	5	10	4	5	0	9
2	22	3	5	8	4	5	0	9
2	22	3	5	8	4	5	0	9
1	21	3	3	6	3	5	0	8
2	19	3	3	6	3	5	0	8
2	20	3	3	6	3	5	0	8

2	18	3	3	6	3	5	5	13
1	19	3	3	6	3	5	0	8
2	19	5	5	10	4	5	0	9
1	18	5	5	10	3	5	0	8
2	14	3	3	6	3	5	5	13
2	19	5	5	10	5	5	0	10
2	15	5	5	10	3	5	0	8
1	16	3	3	6	3	5	0	8
1	17	3	5	8	4	5	0	9
1	20	0	0	0	0	5	0	5
1	16	5	5	10	4	5	5	14
1	20	3	3	6	3	5	0	8
2	13	5	5	10	3	0	0	3
2	16	3	3	6	3	5	0	8
2	18	3	3	6	4	5	0	9
2	18	3	3	6	4	5	0	9
2	13	0	0	0	0	0	0	0
2	18	3	3	6	3	5	0	8
2	26	3	3	6	4	5	0	9
2	17	3	3	6	3	5	0	8
1	19	3	3	6	3	5	0	8
1	17	0	0	0	0	0	0	0
1	18	3	3	6	3	5	0	8
2	25	3	3	6	3	5	0	8
1	24	3	3	6	3	5	0	8
1	23	3	3	6	3	5	0	8
1	21	3	3	6	4	5	5	14
1	18	3	3	6	3	5	0	8
2	16	3	3	6	3	5	0	8
2	21	0	0	0	0	0	0	0
2	17	3	3	6	3	5	0	8
1	20	3	3	6	3	5	0	8
2	13	3	3	6	3	5	0	8
1	13	3	3	6	3	5	5	13
1	13	3	3	6	3	5	5	13
2	21	0	0	0	0	0	0	0
1	17	3	3	6	3	5	0	8
2	22	3	3	6	3	5	0	8
2	22	3	3	6	3	5	0	8
2	18	3	3	6	3	5	0	8

SupportFac	SHAHelpQ	SupHome_2	HelpedMost	ofFMtohelp	pedScore0C	MCProvideratHomeY	MC_TOT_K	_TOT_SUPP
19	3	2	3	2	2	5	16	9
19	3	2	3	2	2	5	16	9
14	1	2	3	1	1	5		
16	3	2	3	0	0	0		
9	3	2	3	0	0	0		
9	3	2	3	1	1	5	13	5
16	3	2	3	1	1	5		
9	3	2	3	2	2	5		
19	3	2	3	2	2	5	11	8
19	3	2	3	2	2	5	11	8
14	4	2	3	1	1	5	16	3
14	3	2	3	0	0	0		
3	3	2	3	0	0	0		
9	3	2	3	0	0	0		
9	3	2	3	0	0	0		
9	3	2	3	0	0	0		
0	3	2	3	0	0	0		
14	3	2	3	1	1	5		
3	4	2	3	0	0	0		
6	3	2	3	1	1	5	15	3
14	4	2	3	2	2	5	23	9
9	3	2	3	1	1	0		
9	3	2	3	1	1	0		
9	2	2	3	1	1	0		
14	5	2	3	1	1	0		
14	4	2	3	1	1	0		
14	3	2	3	1	1	0		
9	4	2	3	2	2	0		
15	3	2	3	2	0	0		
14	3	2	3	2	0	0		
3	4	2	3	2	0	5		
14	4	2	3	1	1	0		
14	2	2	3	0	0	0		
14	4	2	3	0	0	5		
14	4	2	3	2	2	0		
0	4	2	3	0	0	5	17	3
19	4	2	3	1	1	5	17	10
9	3	2	3	1	1	0		
19	4	2	3	1	1	5	15	9
3	3	2	3	1	1	0		
9	4	2	3	1	1	5	18	3
14	4	2	3	1	1	0		
14	5	2	3	1	1	5	23	4
19	4	2	3	1	1	5	20	9
14	2	2	3	1	1	0		
9	4	4	3	2	2	5	14	7

20	4	4	3	1	1	5	15	9
20	4	4	3	1	1	5	15	9
9	3	2	3	1	1	5	16	5
14	4	8	3	4	3	5	17	6
9	5	4	3	2	2	5	16	10
14	3	0	0	1	1	0		
14	4	2	3	1	1	0		
9	3	0	0	3	3	0		
9	4	4	3	2	2	5	14	9
14	4	2	3	3	3	0		
14	5	4	3	2	2	5		
9	4	4	3	2	2	0		
14	4	2	3	1	1	0		
21	3	2	3	2	2	5	20	10
14	3	4	3	2	2	5	11	5
14	4	4	3	2	2	5	19	10
14	3	2	3	1	1	0		
14	3	4	3	2	2	5	15	7
19	3	2	3	1	1	5	12	8
9	3	4	3	2	2	0		
9	3	2	3	1	1	0		
14	4	2	3	2	2	5	18	5
10	4	2	3	1	1	0		0
11	4	4	3	1	1	0		0
16	4	4	3	2	2	5	14	2
9	4	2	3	1	1	5	24	9
19	3	2	3	1	1	5	16	9
6	4	2	3	1	1	5	17	6
19	4	2	3	1	1	5	17	8
19	4	2	3	1	2	5	18	10
19	4	2	3	1	2	5	18	10
14	3	2	3	1	1	0		
14	3	2	3	1	1	5	19	4
16	4	4	3	1	1	0		
8	4	6	3	1	1	5	20	4
18	4	4	3	1	1	0		
6	4	2	3	1	1	0		
23	4	4	3	2	2	5	16	11
16	4	2	3	1	1	0		
14	5	4	3	2	2	0		
14	5	6	3	2	2	0		
19	3	2	3	1	1	5	17	11
6	6	2	3	1	1	0		
9	4	4	3	2	2	5	12	4
23	4	2	3	1	1	5	16	12
18	5	2	3	1	1	0		
14	4	4	3	2	2	5	14	4

13	5	2	3	1	1	0		
14	3	2	3	1	1	0		
23	5	4	3	2	2	5	12	11
23	5	4	3	2	2	5	12	11
12	4	2	3	0	0	0		
3	5	4	3	1	1	0		
3	5	2	3	1	1	0		
14	4	2	3	1	1	0		
14	3	4	3	1	2	5		
14	4	4	3	2	2	0		
21	3	4	3	2	2	5	15	7
21	3	4	3	2	2	5	15	7
18	4	4	3	2	2	5		
18	4	4	3	2	2	5		
21	4	2	3	1	1	5	17	7
23	4	4	3	2	2	5	14	5
18	4	2	3	1	1	0		
9	4	2	3	2	2	0		
5	4	2	3	1	1	5	16	4
19	3	4	3	2	2	5	12	5
0	4	6	3	3	3	5		
16	4	2	3	1	1	5	15	8
23	4	4	3	2	2	5	16	8
16	4	6	3	2	2	5		
15	3	2	3	1	1	5	16	4
15	4	4	3	2	2	5	20	11
18	3	4	3	2	2	5	13	4
18	3	4	3	2	2	5	13	4
17	2	0	0	0	0	0		
17	2	0	0	0	0	0		
0	4	2	3	1	1	0		
15	4	4	3	1	1	0		
14	4	2	3	1	1	0		
20	6	4	3	2	2	5	24	15
15	4	4	3	3	3	5	14	3
15	4	4	3	3	3	5	14	3
22	3	2	3	2	2	5	23	10
9	5	2	3	2	2	0		
19	4	2	3	2	2	5	16	9
22	3	2	3	1	1	5	18	9
21	1	2	3	2	2	5	18	8
14	5	4	3	1	1	0		
23	4	4	3	3	3	5	15	8
10	5	4	3	2	2	5	20	11
18	3	2	3	1	1	5		
19	4	2	3	2	2	0		
14	5	4	3	2	2	5		

13	2	2	3	1	1	0		
14	4	2	3	1	1	0		
14	5	2	3	2	2	5		
17	3	2	3	1	1	0		
13	4	2	3	1	1	5	16	4
18	5	4	3	2	2	0		
17	4	4	3	2	2	0		
23	3	6	3	3	3	5	16	7
11	4	4	3	2	2	5	16	4
16	4	2	3	1	1	0		
18	4	4	3	2	2	0		
14	4	4	3	2	2	5	20	9
14	4	6	3	2	2	5	20	4
23	3	6	3	3	3	5	19	9
17	3	4	3	2	2	0		
17	3	4	3	2	2	0		
14	3	2	3	1	1	0		
14	4	4	3	2	2	0		
15	5	4	3	2	2	5	26	6
15	5	4	3	2	2	5	26	6
15	3	6	3	1	1	0		
14	4	4	3	2	2	0		
19	2	2	3	1	1	5	16	9
19	2	2	3	1	1	5	16	9
14	4	2	3	0	0	0		
14	4	4	3	2	2	0		
10	5	2	3	1	1	5	18	5
21	4	4	3	2	2	5	17	10
19	4	4	3	2	2	5	18	9
19	4	4	3	2	2	5	17	9
19	4	4	3	2	2	5	17	9
17	4	4	3	2	2	5	18	10
17	4	4	3	2	2	5	17	4
16	4	2	3	1	1	0		
14	4	4	3	2	2	5		
14	4	4	3	2	2	0		
14	2	4	3	2	3	5	10	4
18	4	4	3	2	3	0		
14	4	6	3	2	2	0		
20	4	4	3	2	2	5	18	10
14	2	2	3	1	1	0		
19	4	2	3	1	1	0		
17	4	2	3	1	1	5	22	10
17	4	2	3	1	1	5	22	10
14	5	4	3	1	1	0		
14	3	4	3	2	2	0		
14	3	2	3	2	2	0		

19	4	6	3	3	3	5	18	8
14	4	4	3	2	2	0		
19	4	2	3	1	1	0		
18	4	4	3	2	2	0		
19	4	4	3	2	2	5	12	10
20	4	2	3	1	1	0		
18	2	2	3	2	2	0		
14	4	2	3	1	1	0		
17	3	2	3	1	1	0		
5	4	2	3	1	1	0		
24	4	4	3	1	1	5	20	10
14	4	6	3	2	2	5		
13	4	4	3	1	1	0		
14	0	2	3	1	1	5	17	5
15	3	4	3	2	2	5	16	4
15	3	4	3	2	2	5	16	4
0	5	2	3	1	1	0		
14	2	4	3	2	2	0		
15	3	2	3	1	1	0		
14	3	2	3	1	1	0		
14	4	4	3	2	2	0		
0	5	2	3	2	2	0		
14	4	2	3	1	1	0		
14	4	4	3	1	1	0		
14	1	2	3	1	1	0		
14	3	2	3	2	2	5	17	7
20	2	2	3	1	1	5	19	9
14	4	4	3	1	1	0		
14	3	2	3	1	1	0		
0	5	4	3	1	1	0		
14	3	4	3	1	1	0		
14	3	4	3	1	1	0		
14	3	6	3	1	1	0		
19	3	2	3	1	1	5	17	10
19	3	2	3	1	1	5	17	10
0	6	4	3	1	1	5	17	5
14	4	4	3	1	1	0		
14	4	2	3	1	1	0		
14	4	2	3	1	1	0		
14	4	2	3	1	1	5	15	4

MC_TOT_A:ed_Q5.KM(		M_SupHomeTOTAL38
4	3	29
4	3	29
		13
		6
		6
4	0	26
	3	19
		16
4	2	27
4	2	27
4	2	25
		6
		6
		6
		6
		6
		6
		15
		7
3	2	24
4	8	33
		7
		7
		6
		9
		8
		7
		9
		6
		6
		15
		8
		5
		15
		9
4	6	28
4	4	30
		7
4	6	29
		7
3	3	27
		8
4	4	30
3	7	30
		6
4	2	28

3	3	27
3	3	27
4	6	29
4	2	30
4	2	31
		4
		8
		6
4	4	30
		10
	2	21
		9
		8
4	5	31
4	2	27
4	3	31
		7
4	2.5	28
4	1.5	26
		8
		7
3	6	30
		8
		8
4	4.5	29
4	4.5	31
4	3.5	28
4	3.5	29
4	5.5	30
4	4	31
4	4	31
		7
4	4	27
		8
4	1.5	27
		8
		8
4	2	30
		8
		10
		10
4	6	30
		10
4	6	29
4	2	30
		9
4	3	28

		9
		7
4	7	33
4	7	33
		7
		9
		9
		8
	4	20
		9
4	3	28
4	3	28
	3.5	21
	3.5	21
4	2.5	29
4	2.5	29
		8
		9
4	2.5	28
4	1.5	27
		18
4	2	27
4	2.5	30
	3	21
4	3	27
4	20	33
4	3	27
4	3	27
		2
		2
		8
		8
		8
4	2.5	34
4	6	30
4	6	30
4	2	30
		10
4	6	31
4	3	28
4	3.5	27
		9
4	2	29
4	2.5	32
		15
		9
		18

		6
		8
	2.5	22
		7
4	2	27
		10
		9
4	3	30
4	2	28
		8
		9
4	6	31
4	6	30
4	3.5	30
		8
		8
		7
		9
4	6	33
4	6	33
		7
		9
2	2	24
2	2	24
		7
		9
4	2.5	30
4	1.5	30
4	2.5	30
4	3	30
4	3	30
4	1.5	30
4	0.75	28
		8
		17
		9
4	2	26
		10
		9
3	2	29
		6
		8
4	5	31
4	5	31
		9
		8
		8

4	0.75	30
		9
		8
		9
4	1.5	29
		8
		7
		8
		7
		8
4	1.5	29
		17
		8
3	2	23
4	3	28
4	3	28
		9
		7
		7
		7
		9
		10
		8
		8
		5
2	2	26
4	2.5	27
		8
		7
		9
		7
		7
		7
4	2	28
4	2	28
4	2	30
		8
		8
		8
4	3	26

PID
S.No
Q4.BirthWeightGrams
BWcat
Q5.SexM1F2O0
Q2.PlaceofBirthDHTH1CHC2PHC3Pvt4Home5OD5
placebirth
Q8.AdmittedWhereTH1CHC2PHC3Pvt4Referredout5athome0
Admitted
BirthAdmission
AvgKnow_HCP
AvgAtt
AvgSkill
babystatusKMC2Well1Sick2greenNullatBirth
HCPCompetency_Point1_170
HCPComp_Point1
HCPComp_Point2_170
HCPComp_Point2
AverageHCPCompetency
AvgHCPComp
Point1_HFacPrep_100
Point2_HFacPrep_100
AverageHealthFacilityPreparedness
Q1.DOB
M_Q11.ii.DateKMCstartedMothers
Q11.iii.KMCWherefromMotherTH1CHC2PHC3Pvt4Home5DHRO6
M_Q11.iii.KMCWhereTHDH1CHC2PHC3Pvt4Home5WHO_Mother
WHO_KMCD1hours
DAY_KMC_Initiated
WHO_KMClt24hours
days_KMC3_RR
KMC24hours
KMC_7day
WHO_BFlt24Discharge
M_KMCInitiatedFac1Home0
KMC_initiationRR
Day_KMC3_RR
DAYS_KMC3
KMC24_LR
KMC28
KMC_EBF_24
kmc7_LR
KMC_EBF_7
KMC28_LR
WHO_StatusSHH1RO2Died3LAMA4
WHO_KMC_D7
WHO_EBF_D7

WHO_KMC_D28hours
WHO_EBF_day28
M_DateofInterview
M_Q13.i.KMCendeddateifcontinuingDateofinterviewwasput
M_NoofDaysKMCgive
M_Q16.FollowuptohospitalY1N0
M_Qs22.i.DInterviewEBFY1Anyother0
M_Q6.HealthStatusDayofInterviewWell1Sick0
Q9.Durationdayscolouredifnormalstay
Hospcat
hosp_cat
Q17.M_Ageyrs
Q18.MEducationYears
Meducat
M_Occ_Skilled1Unskilled2Business3Homemaker4
Moccup_cat
M_Edu_Nil11526123gt134cationcat
Q19.MOccupation1.Skilled2unskilled3business4HW
Q21.ii.M_AgeofSpouseyrs
Q20.M_EducationSpouse
M_SpouseEdu_Nil11526123gt134cationcat
Q21.i.M_OccSpouse
M_SpouseOcc
Q24.i.M_NoofChildren
M_NoChildrenPrimi112gt23
M_Knowledge_TOTAL30
M_HelpedIniTotQ127Y10HCP122344566
M_CounselledTOTQ137BA10if122if344if566
M_SupIniKMC_14
M_HelpedMostTotQ14BI1or000HCP12234354
M_Q15.KMCKitY5N0
FKMC_HospQ18.Y5No0Grey_noFKMC
M_SupMaintenanceHospital_Tot15
M_SupportFac_29
M_ASHAHelpQ17_6
M_Q18TotSupHome_2for1person
M_SuppHomeHelpedMostScore_0Nil3gt1
M_NoofFMtohelp_Actual
NoFMHelpedScore001122gt33
FKMCProvideratHomeY5N0
FKMC_TOT_K30
FKMC_TOT_SUPPORT18
FKMC_SupportCat51NAseeColBR2lt4.535941011.255gt11.5
FKMC_TOT_Att4
FKMC_Att_catNA112233445
MorFKMCreported_Q5.KMCdurationdayhrs
FKMC_ScoreforHrKMC00NA22h334h456h5gt7h6

M_SupHomeTOTAL38

Participatnt identiicaito

